# Dual RNA sequencing reveals the transcriptomic and cellular response of *Cannabis sativa* to infection by the fungal pathogen *Sclerotinia sclerotiorum*

**DOI:** 10.1038/s41598-026-47998-2

**Published:** 2026-04-16

**Authors:** Natalie L. Cale, Rylee E. Swiderek, Philip L. Walker, Dylan J. Ziegler, Brigo Castillo, Sean M. Robertson, Olivia Wilkins, Mark F. Belmonte

**Affiliations:** 1 Department of Biological Sciences, University of Manitoba, Winnipeg, MB Canada; 2 Morden Research and Development Centre, Agriculture and Agri-Food Canada, Morden, MB Canada; 3 Department of Biology, University of Victoria, Victoria, BC Canada

**Keywords:** Microbiology, Molecular biology, Plant sciences

## Abstract

Known to infect more than 600 plant species worldwide, *Sclerotinia sclerotiorum* is a necrotrophic fungal pathogen, and the causative agent of white mold. With recent infection reports documented across North America, *Cannabis sativa* is known to be susceptible to *Sclerotinia* infection. Resulting from legal constraints associated with *C. sativa*, little is known about the *Cannabis-Sclerotinia* pathosystem, particularly in how the plant responds to pathogen attack at the cellular and molecular levels. Our anatomical study revealed initial infection and degradation of the epidermis and cortical parenchyma, followed by widespread infection of the vascular phloem. Dual RNA sequencing of the *C. sativa* cola provided a detailed transcriptomic profile of this pathosystem directly at the site of infection over time. Differential gene expression analysis revealed large-scale transcriptional shifts resulting from rapid infection. Gene ontology term enrichment identified processes associated with plant defense and signal transduction cascades during *C. sativa* infection while processes associated with redox control and sugar catabolism were enriched in the *S. sclerotiorum* pathogen. Taken together, this study revealed transcriptional reprogramming in both the host plant and fungal pathogen associated with floral infection in space and time.

**Supplementary Information:**

The online version contains supplementary material available at 10.1038/s41598-026-47998-2.

## Introduction

*Cannabis sativa* L. (hereafter referred to as *Cannabis or C. sativa)* is believed to have originated in Central Asia^1,2^ and remains one of the most widely cultivated, yet controversial plants grown worldwide^3^. Grown for its fibre, medicinal, and psychoactive properties, *Cannabis* quickly spread throughout Asia and Europe, and today is grown and sold internationally both legally and illegally^4^. *C. sativa* is a diploid (2n = 20) and dioecious flowering plant species, one of few plants to use an XY chromosomal system of sex differentiation, with a male determining Y ^2,5^. Female inflorescences, specifically the bracts of the flowers, are densely covered in resin-containing secretory glandular trichomes, the site of cannabinoid and terpene biosynthesis and storage^6–8^. Δ^9^-tetrohydrocannabinolic acid (THCA) and cannabidiolic acid (CBDA) are examples of psychoactive and non-psychoactive cannabinoids found within the resin of *C. sativa* secretory glandular trichomes. Along with high levels of mono- and sesquiterpenes which impart scent and flavour characteristics, the composition of this metabolite-rich resin greatly influences *Cannabis* consumer preference^6,9,10^. In other plant species, terpenes are known to serve various roles ranging from attracting beneficial pollinators, to serving as chemical deterrents to herbivores, though how these secondary metabolites specifically benefit *C. sativa* has yet to be explored^11,12^. As the female inflorescence is the region of highest glandular trichome density, and thus cannabinoid/terpene-containing resin, it is this structure that is harvested for retail drug sale and is the focus of this study.

In October of 2018, Canada became the second country to legalize *C. sativa* for non-medical use and retail sale^13,14^, and as of March 2024, its registered growing area in Canada was reported at 1.39 and 6.24 million m^2^ for indoor and outdoor growing area, respectively^15^. Recently, emerging diseases of *C. sativa* have been reported because of this extensive cultivation. Reports of *S. sclerotiorum*, the causal agent of white mold, have been described in field and greenhouse settings across North America, and has resulted in the pathogen being deemed an emerging concern for both the medicinal *Cannabis* and industrial hemp industries^16–21^. Known to infect more than 600 plant species worldwide including agricultural and horticultural crops, ornamentals, trees/shrubs and weed species, *S. sclerotiorum* is responsible for devastating yield losses^22–25^. Although losses in yield vary considerably based on geographic location and species, losses in favourable conditions for infection are often reported at 20–35%, although losses over 50% and up to 80–100% have been documented^26,27^. *S. sclerotiorum* infection is difficult to control largely due to its rapid and aggressive disease progression along with its capacity for long-term persistence in the soil in the form of sclerotia. In appropriate conditions, these sclerotia may germinate myceliogenically or carpogenically; resulting in direct host infection via mycelia or by airborne ascospores released by apothecia, respectively^22,27^. Studies conducted in susceptible crop species such as *B. napus* and *Helianthus annulus* (sunflower), among others, have revealed that *S. sclerotiorum* uses simple and complex appressoria in tandem with a variety of cell wall degrading enzymes, oxalic acid, and other pathogenic effectors to penetrate and degrade host plant tissues, ^27-60 -30^. Ultimately, this leads to cell death, necrotic lesion formation, and eventually systemic infection and plant death^31,32^. While the specific lifecycle and interactions between *S. sclerotiorum* and many crop hosts have been well-documented, the lifecycle and cellular and molecular interactions of the *C. sativa* – *S. sclerotiorum* pathosystem has yet to be explored. Infection reports detailing symptoms of *S. sclerotiorum* infection in *C. sativa* have highlighted the development of friable tan/brown necrotic cankers and lesions developing on the crown, along the stem, and within the inflorescence of plants^16,17,21^. Also documented was the presence of white mycelium and sclerotia present at the site of the lesion, as well as within the pith cavity of the stem. Despite the recent publication of these infection reports, the interaction between *C. sativa* and *S. sclerotiorum* has yet to be described at the cellular and molecular levels.

Plants have evolved complex defense mechanisms to defend against pathogenic attack. Upon detection of specific pathogen-derived molecules, plants respond through the activation of innate immune pathways^33,34^. Such molecules include pathogen- or damage-associated molecular patterns (PAMPs and DAMPs, respectively) detected by pattern recognition receptors (PRRs), specifically receptor like protein kinases (RLKs), or through detection of pathogenic elicitors by nucleotide binding leucine rich repeat (NLR) receptors^35^. While recognition of PAMPs/DAMPs by PRRs initiates pattern triggered immunity (PTI), pathogenic elicitor detection via NLR receptors results in the initiation of effector triggered immunity (ETI). Although PTI is generally regarded to confer immunity against non-adapted pathogens and ETI, through a more robust immune response, against host-adapted pathogens, elaborate crosstalk between pathways has been previously observed with co-induction having led to increased pathogen resistance^36,37^. Following pathogen recognition and immune activation, early defense responses include cellular calcium import and signal transduction cascades, reactive oxygen species (ROS) burst, and phytohormone signalling that lead to defense-related gene induction and induced resistance responses^32,35,38^. These defense pathways include systemic acquired resistance (SAR), associated with salicylic acid (SA), and induced systemic resistance (ISR) associated with ethylene (ET) and jasmonic acid (JA)^39^. Induction of SA-dependent SAR results in increased systemic pathogenesis related (PR) protein expression. Conversely, ISR induction, often activated by beneficial microbe colonization, results in the adoption of a primed defense state allowing for more rapid defense responses upon subsequent pathogen challenge^40^.

In the present work, we studied the transcriptomic response of the *C. sativa* cola to infection with *S. sclerotiorum* across a seven-day period and complemented these experiments with a detailed anatomical study of the infection process. RNA sequencing results revealed large transcriptomic shifts occurring in both the host plant and fungal pathogen. While genes involved in redox buffering and carbohydrate metabolism were enriched in *S. sclerotiorum*, *C. sativa* responded to infection through initiating facets of the plant defense response including hormone and cellular signalling cascades, the SAR response, and cell wall reinforcement activities. Host and pathogen transcriptional reprogramming aligned with degradation of host cortical and vascular phloem tissues. Together, these results serve as the first transcriptomic and cellular descriptions of *S. sclerotiorum* infection of *C. sativa.*

## Results

### *S. sclerotiorum* initiates rapid infection in *C. sativa* floral tissue

**Fig. 1:**
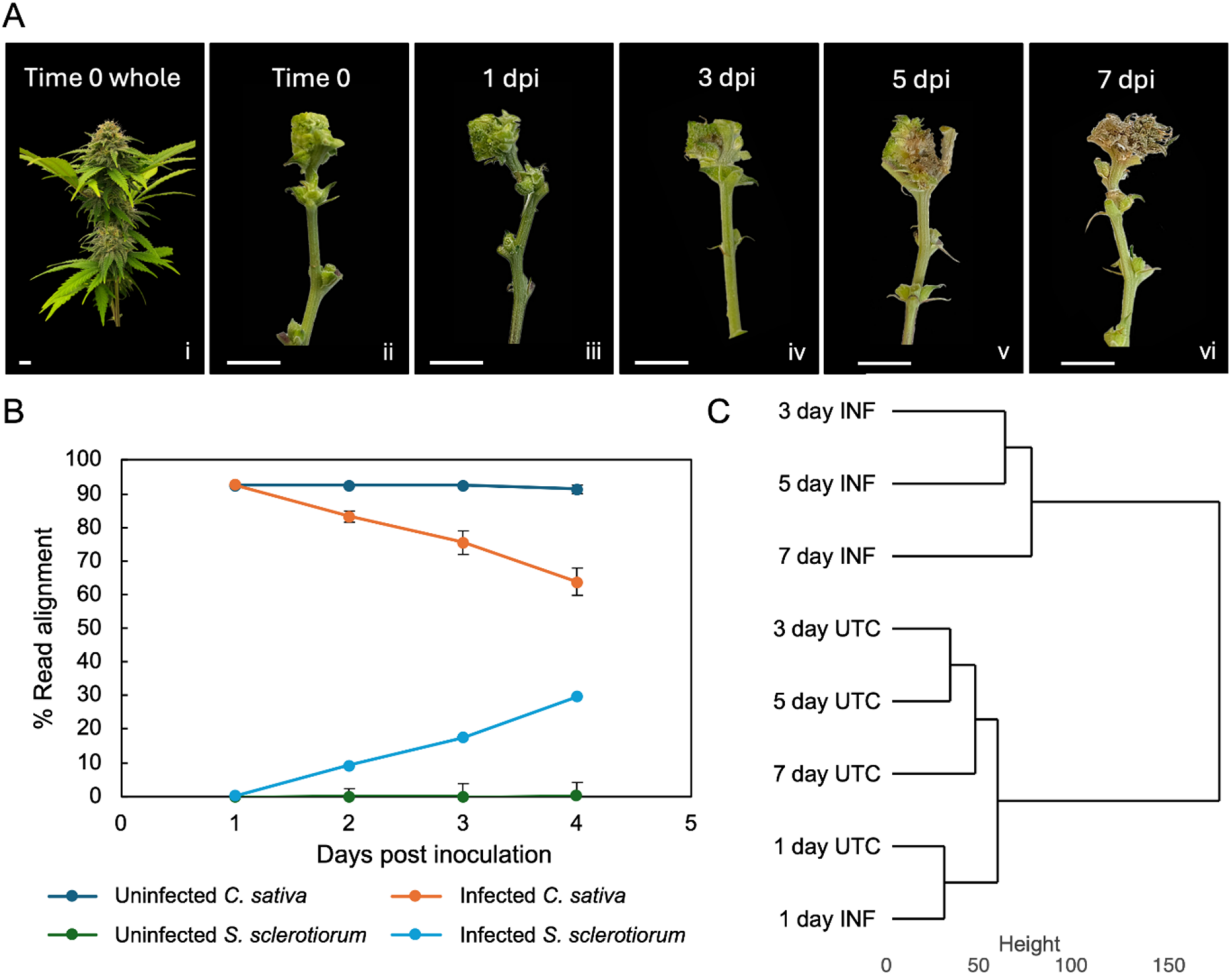
*S. sclerotiorum* infection of the *C. sativa* cola. (**A**) Symptom progression of *S. sclerotiorum* infection of the *C. sativa* cola up to seven days post inoculation (dpi). Whole cola (i) pictured next to trimmed cola (ii), both at time zero. Trimmed infected colas pictured at 1-, 3-, 5-, and 7 dpi indicated as iii, iv, v, and vi, respectively. Scale bar = 1 cm. (**B**) Percent alignment of RNA sequencing reads from infected and uninfected control samples to both the *C. sativa* and *S. sclerotiorum* genomes. Error bars correspond to standard error. (C) Dendrogram of *C. sativa* samples based off hierarchical clustering of the top 10,000 most variable genes. Height corresponds to Euclidean distance between clusters. INF = infected, UTC = untreated control.

First, we performed *S. sclerotiorum* infection assays of the *C. sativa* cola to better understand disease progression over time (Fig. 1). At one day post inoculation (dpi) no external disease symptoms were visible (Fig. 1A, i-iii). At 3 dpi, we first observed floral tissue necrosis at the site of inoculation (Fig. 1A, iv). By 5 dpi, necrosis was observed throughout the inoculated inflorescence and had extended to the inflorescence axis, nearing the main stem of the cola (Fig. 1A, v). Finally, at 7 dpi, necrosis had become widespread, affecting neighbouring inflorescences of the cola and had extended down into the main stem axis (Fig. 1A, vi). Infected necrotized tissues were pale brown in colour, and friable. Necrotic tissue present in the interior of the cola was soft and water-soaked, while necrotic tissue found towards the exterior of the cola was dry and brittle. Alignment of RNA sequencing reads to *S. sclerotiorum* increased in infected samples as infection time progressed, while the opposite trend was observed for reads aligned to the *C. sativa* genome (Fig. 1B). This finding was further supported by qPCR results quantifying relative fungal load (Supplementary Figure S1). Targeting *S. sclerotiorum* 18 S rDNA, data revealed *S. sclerotiorum* became more abundant in the *C. sativa* cola during the seven day infection process.

Global shifts in gene expression were observed in both the host plant and fungal pathogen as *S. sclerotiorum* initiated infection in *C. sativa*. Hierarchical clustering the top 10,000 most variably expressed *C. sativa* genes revealed that treatments clustered together based on infection status with the exception of the 1 dpi timepoint which remained clustered with uninfected samples (Fig. 1C). These results were supported by principal component analysis (PCA) of individual samples which revealed that the largest source of variation in our data was attributed to infection status (Supplementary Figure S2). Furthermore, infected samples clustered into distinct groups based on time post inoculation, highlighting large shifts in gene activity as infection progressed across the seven-day infection period. Similarly, hierarchical clustering of the top 1000 most variably expressed *S. sclerotiorum* genes revealed the 1 dpi timepoint to cluster with in vitro grown *S. sclerotiorum*, while all other infection timepoints clustered distinctly (Supplementary Figure S3). PCA of individual samples directly supported hierarchical clustering results (Supplementary Figure S4). We validated the RNA sequencing results by comparing the relative expression of the SAR marker gene *PATHOGENESIS RELATED PROTEIN 1* (*PR-1*) using RT-qPCR. Data show *PR1* accumulates at similar levels regardless of the method used to evaluate its activity (Supplementary Figure S5).

### *S. sclerotiorum* rapidly infects *C. sativa* tissues and preferentially infects phloem tissues

**Fig. 2:**
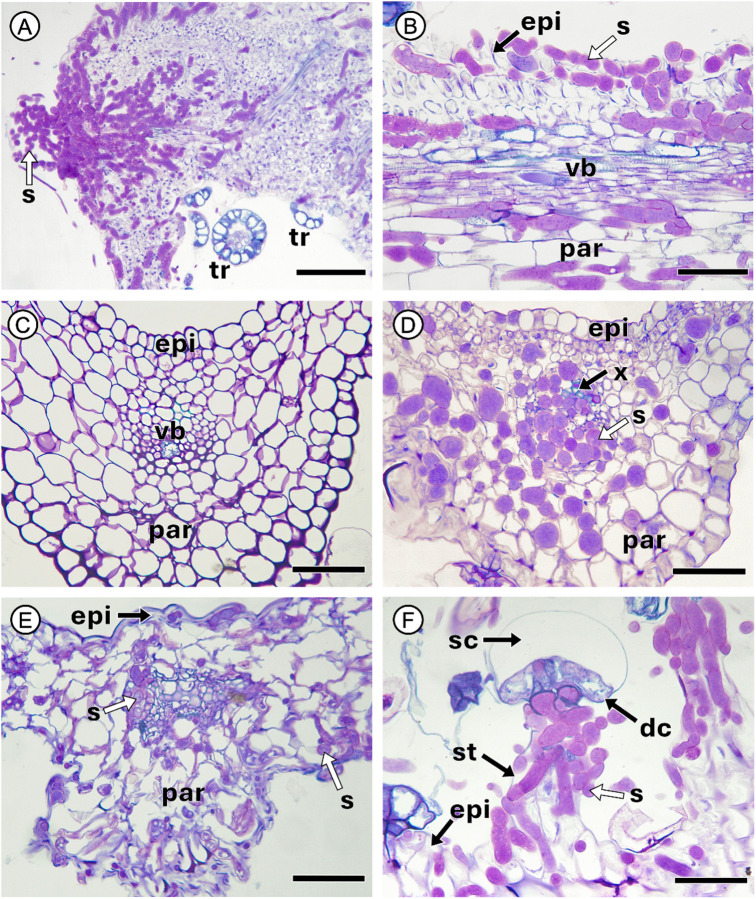
*S. sclerotiorum* infection of the *C. sativa* inflorescence and infection progression in reduced leaves. (A) Longitudinal section of the *S. sclerotiorum* inoculation site (s, white arrow) three days post inoculation. Trichomes (tr) are visible throughout the inflorescence. Scale bar = 100 μm. (B) Longitudinal section of the reduced leaf within the inflorescence. *S. sclerotiorum* hyphae (s, white arrow) found in epidermis (epi), parenchyma (par), and vascular bundle (vb) three days post inoculation. Scale bar = 50 μm. (C) Cross section of uninfected reduced leaf. Vascular bundle (vb), epidermal cells (epi) and parenchyma (par). Scale bar = 50 μm. (D) Cross section of reduced leaf three days post inoculation. *S. sclerotiorum* hyphae (s, white arrow) present throughout vascular bundle and parenchyma (par). Tracheary cells of the xylem (x) remain intact. Scale bar = 50 μm. (E) Cross section of reduced leaf seven days post inoculation. *S. sclerotiorum* hyphae (s, white arrow) present in epidermis (epi), parenchyma (par), and vascular bundle. Xylem (x) tracheary cells remain intact. Scale bar = 50 μm. (F) *S. sclerotiorum* infection of the stalk (st) and disk cells (dc) of a glandular trichome seven days post inoculation. Secretory cavity (sc) and cuticle are still intact. Scale bar = 50 μm.

**Fig. 3:**
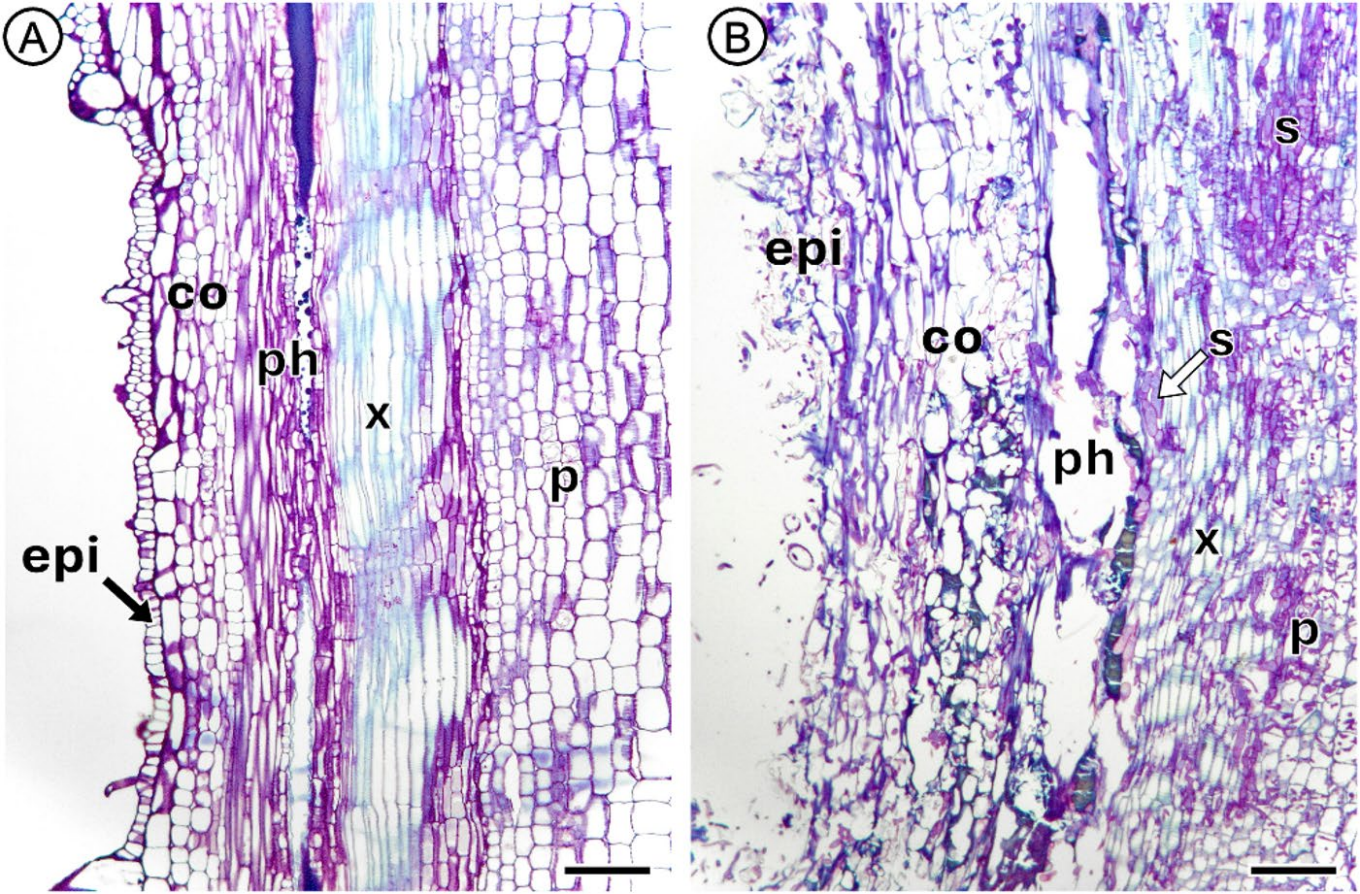
Longitudinal sections of the *C. sativa* inflorescence stem uninfected versus seven days post inoculation with *S. sclerotiorum*. (**A**) Longitudinal section of an uninfected *C. sativa* inflorescence stem. Epidermis (epi), cortex (co), phloem (ph), xylem (x), and pith (p) are labelled accordingly. Scale bar = 100 μm. (**B**) Longitudinal section of a *C. sativa* inflorescence stem infected with *S. sclerotiorum* seven days post inoculation. Extensive tissue degradation is apparent across the epidermis (epi), cortex (co), phloem (ph), and pith (p). The xylem (x) remains relatively intact when compared to other tissue layers. *S. sclerotiorum* (s) is present throughout the tissue layers of the stem. Scale bar = 100 μm.

To better understand the interaction between *S. sclerotiorum* and *C. sativa* we tracked fungal infection of the cola at the cellular level directly from the site of inoculation (Fig. 2A). At 3 dpi, the inoculation site was clearly visible, as was the extension of fungal hyphae as *S. sclerotiorum* began to infect host floral tissue. At this timepoint, sectioning of reduced leaves proximal to the inoculation site revealed the presence of fungal hyphae along the surface of the epidermis as well as within epidermal cells, palisade mesophyll and general parenchymatic tissues, and phloem tissue of the vascular bundle (Fig. 2B). Xylem tissues remained relatively untouched whereas the phloem showed extensive colonization by the fungus as compared to uninfected reduced leaves (Fig. 2B-D). While the presence of hyphae was found throughout the reduced leaf, minimal plant cell wall degradation was visible. By 7 dpi, the *C. sativa* reduced leaf showed severe degradation of all tissue layers apart from the xylem (Figs. 3E). Although still structurally intact, *S. sclerotiorum* hyphae were visible throughout the xylem at this timepoint. The *C. sativa* stalked glandular trichomes of the inflorescence were also infected at this timepoint (Fig. 2F).

In the floral stem of the plant, *S. sclerotiorum* infection resulted in extensive host tissue degradation (Fig. 3). The floral stem of *C. sativa* is divided into distinct cell and tissue layers including the epidermis, cortex, phloem, xylem and pith (Fig. 3A). Extensive tissue degradation was revealed in infected floral stem tissues where *S. sclerotiorum* hyphae were abundant in the epidermis, cortex, vascular tissues, and pith of the *C. sativa* stem at 7 dpi (Fig. 3B). Most heavily degraded was the phloem, where cell walls had collapsed entirely, leading to the occurrence of regions of open space in areas where the fungus had used the phloem to travel further into the main stem of the plant. Although hyphae were also present within the xylem tracheids; degradation of this tissue layer was limited.

### Differential gene expression analysis reveals the induction of *C. sativa* defense responses and altered terpenoid production at the mRNA level by *S. sclerotiorum*

**Fig. 4:**
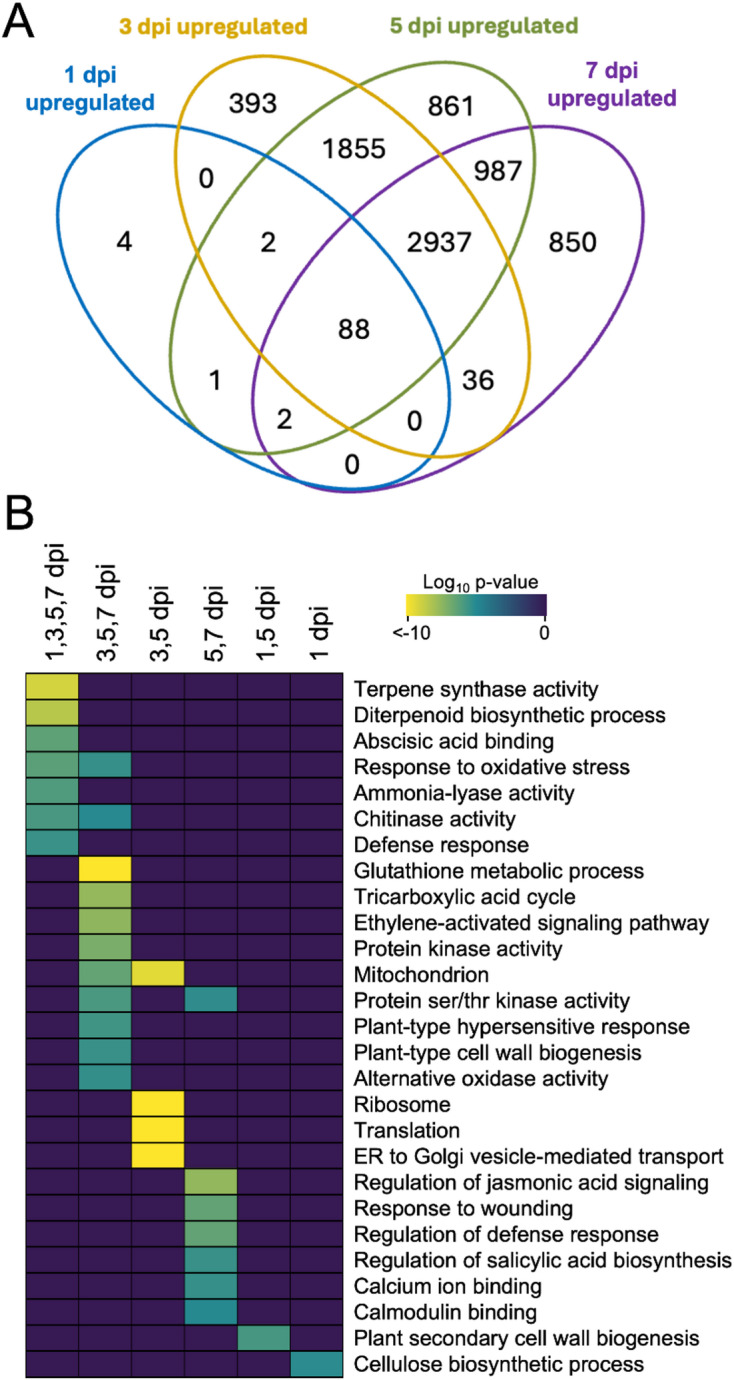
Upregulated differential gene expression of *C. sativa* infected with *S. sclerotiorum* over a seven-day infection period. (**A**) Venn diagram of significantly upregulated differentially expressed gene sets (FDR < 0.05) in response to infection. (**B**) Heatmap of significantly enriched GO terms (FDR < 0.01) resulting from timepoint-specific and shared subsets. A brighter yellow colour indicates greater statistical significance. dpi = days post inoculation.

Next, we carried out differential gene expression analysis to better understand how *C. sativa* responds to *S. sclerotiorum* at the mRNA level (Fig. 4). The largest number of up-regulated differentially expressed genes (DEGs) in *C. sativa* were found at the intersection of 3-, 5- and 7 dpi, and of 3- and 5 dpi, with 2937 and 1855 genes, respectively (Fig. 4A). Specific to each timepoint, 4 genes were upregulated at 1 dpi, 393 genes at 3 dpi, 861 genes at 5 dpi, and 850 genes at 7 dpi. To better understand the biological and molecular processes associated with these gene sets, we conducted a GO enrichment analysis (Fig. 4B). Enriched at all timepoints were GO terms associated with terpene biosynthesis (terpene synthase activity and diterpenoid biosynthetic process), plant stress responses (abscisic acid binding and response to oxidative stress), plant defense, and chitinase activity. Specific to 3- and 5 dpi, we observed enrichment of GO terms associated with protein synthesis/transport (translation and endoplasmic reticulum to Golgi vesicle-mediated transport). Shared between 5- and 7 dpi, were GO terms involved in hormone signalling (regulation of jasmonic acid signalling and regulation of SA biosynthesis), response to wounding, and calcium/calmodulin signalling. More generally, shared between 3-, 5-, and 7 dpi were terms pertaining to oxidative stress responses (glutathione metabolic process and hypersensitive response), ethylene signalling, and protein kinase and protein ser/thr kinase activity. In our data, we identified a larger number of enriched GO terms shared between infection time points while few GO terms were enriched at specific timepoints. All GO enrichment and differential gene expression analysis results and associated P-values are provided in the supporting information (Supplementary Material 1).

**Fig. 5:**
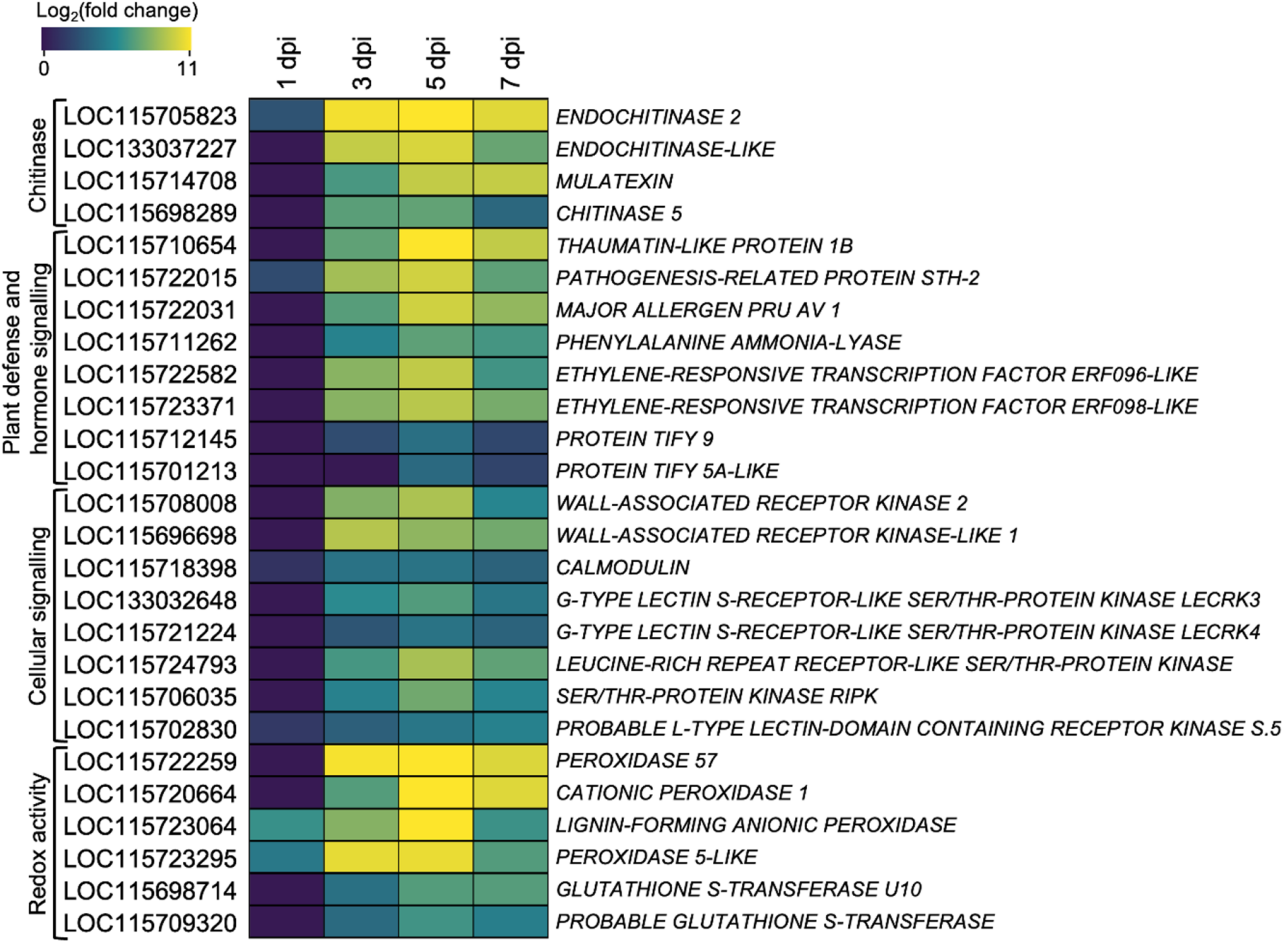
Heatmap of significantly differentially expressed *C. sativa* genes (FDR < 0.05) belonging to enriched GO terms following inoculation with *S. sclerotiorum*. Brighter yellow colour indicates a greater fold change in expression compared to uninfected plants. dpi = days post inoculation.

In response to infection, we uncovered genes encoding chitinases and endochitinases to be upregulated in *C. sativa* (Fig. 5). Those with highest fold changes in response to infection at 5 dpi included *ENDOCHINTINASE 2* (*LOC115705823*; 1982-fold) and *ENDOCHITINASE-LIKE* (*LOC133037227*; 1074-fold). Also seen to be highly upregulated at 5 dpi were genes involved the plant SAR response, including *PATHOGENESIS RELATED* (*PR*) genes. These genes included *THAUMATIN-LIKE PROTEIN 1B* (*PR-5; LOC115710654*; 3487-fold), *PATHOGENESIS-RELATED PROTEIN STH-2* (*PR-10a; LOC115722015;* 917-fold) and *MAJOR ALLERGEN PRU AV 1* (*PR-10; LOC115722031*; 899-fold). Additionally, we observed the induction of genes involved in JA/ET hormone signalling. *JASMONATE-ZIM DOMAIN* (*JAZ*) protein genes *JAZ5* and *JAZ8* in addition to *ETHYLENE RESPONSIVE FACTORs* (*ERF*) *ERF096* and *ERF098* were also significantly differentially expressed. Furthermore, we uncovered the notable upregulation of numerous RLK genes that included *WALL ASSOCIATED RECEPTOR KINASE 2* (*WAK2; LOC115708008*) and *WALL ASSOCIATED RECEPTOR KINASE-LIKE 1* (*WAKL1; LOC115696698*) which both exhibited a 500-fold increase in expression, in addition to various other serine/threonine RLKs that include *G-TYPE LECTIN S-RECEPTOR-LIKE SERINE/THREONINE-PROTEIN KINASE 3* (*LECRK3; LOC113032648*) and *LECRK4* (*LOC115721224*). Finally, a number of peroxidases in *C. sativa* were also found to be highly upregulated in response to *S. sclerotiorum*. These peroxidases included *PEROXIDASE 57* (*PER57; LOC115722259*), *CATIONIC PEROXIDASE 1* (*LOC115720664*), *LIGNIN-FORMING ANIONIC PEROXIDASE* (*LOC115723064*), and *PEROXIDASE 5-LIKE* (*LOC115723295*) with fold changes at 5 dpi of 9710, 2868, 2375, and 1470, respectively.

We were next interested in understanding the genes and biological processes that were downregulated in *C. sativa* in response to infection with *S. sclerotiorum* (Supplementary Figure S6). The largest number of down-regulated genes were shared between 3-, 5- and 7 dpi (2866 genes; Supplementary Figure S6A). Large numbers of shared DEGs were also observed between 3- and 5 dpi (1458 genes) and 5- and 7 dpi (1179 genes). Specific to each timepoint, we identified 850 DEGs at 7 dpi, 1540 DEGs at 5 dpi, 512 at 3 dpi, and interestingly, 0 at 1 dpi. GO enrichment revealed enrichment of terms associated with photosynthesis, cellular development, and terpene synthesis (diterpenoid biosynthetic process and terpene synthase activity) were shared between 3-, 5-, and 7 dpi (Supplementary Figure S6B). Abscisic acid biosynthesis was enriched in gene sets shared between 3- and 5- dpi while biological processes associated with hormone activity like jasmonic acid biosynthesis and cytokinin signalling were specific to gene sets at 3 dpi.

**Fig. 6:**
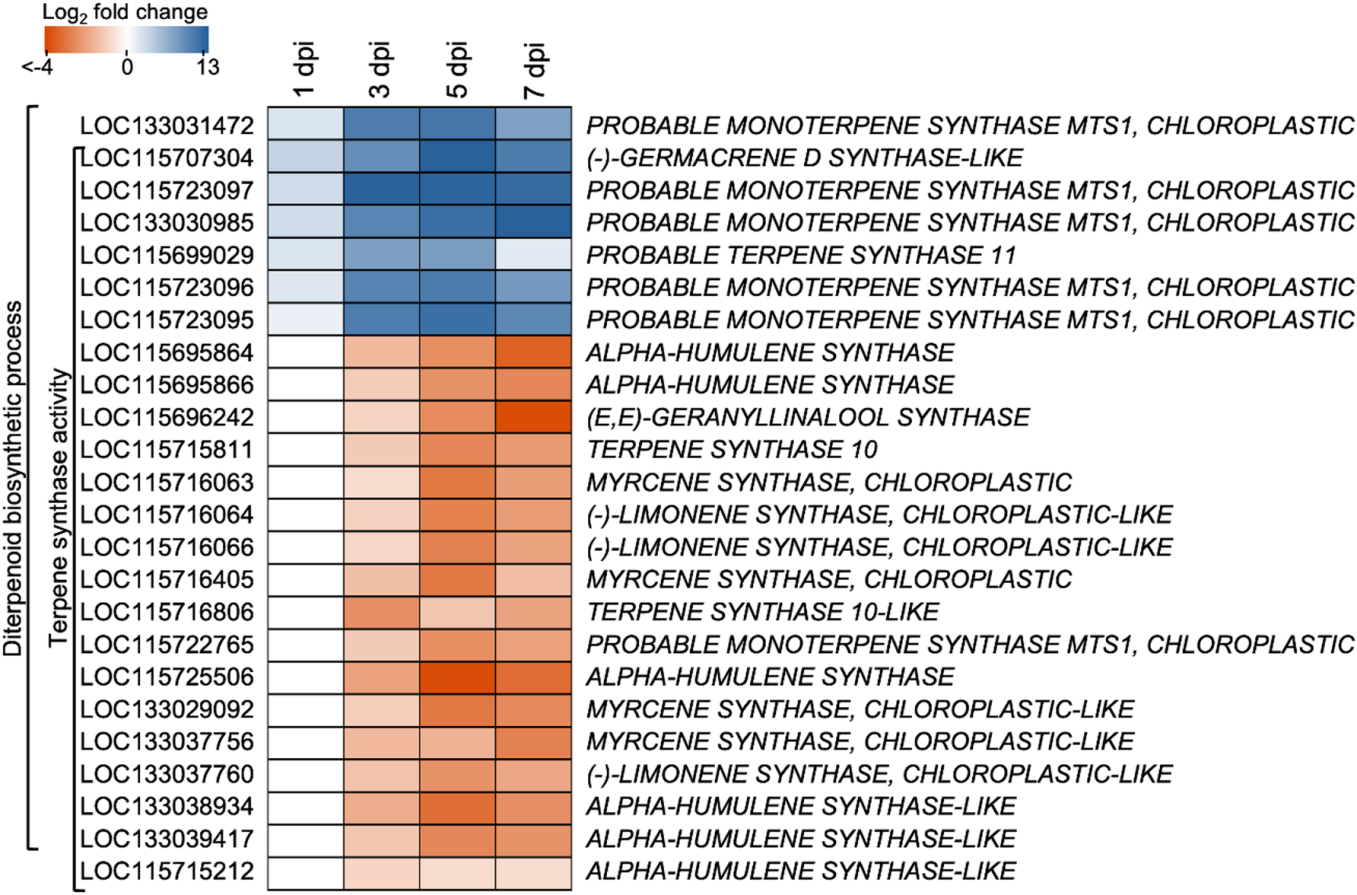
Heatmap of significantly up- and downregulated *C. sativa* genes of the diterpenoid biosynthetic process and terpene synthase activity in response to *S. sclerotiorum* (FDR < 0.05). Colour corresponds to log_2_fold change, where a saturated orange colour corresponds to a greater log_2_fold change downregulation, and a saturated blue colour corresponds to a greater log_2_fold change upregulation. dpi = days post inoculation.

While terpene/diterpenoid biosynthesis GO terms were enriched in both up and downregulated gene sets, the specific genes involved in either GO term were unique (Fig. 6). Upregulated in response to *S. sclerotiorum* infection were *C. sativa* terpene synthases that included *MONOTERPENE SYNTHASE MTS1* (*LOC133031472*,* LOC115723097*,* LOC133030985*,* LOC115723096*,* LOC115723095*) and *(-)-GERMACRENE D SYNTHASE-LIKE* (*LOC115707304*). Both genes were very highly upregulated with fold changes seen as high as 6361 at 7dpi (*LOC13303985*) and 6400 at 5dpi (*LOC115707304*). Conversely, genes downregulated in response to infection included *ALPHA-HUMULENE SYNTHASE* and -*SYNTHASE-LIKE* (*LOC115695864*,* LOC115695866*,* LOC115725506* and *LOC133038934*,* LOC133039417*,* LOC115715212*), *(E-E)-GERANYLLINALOOL SYNTHASE* (*LOC115696242*), *(-)-LIMONENE SYNTHASE* (*LOC115716064*,* LOC115716066*,* LOC133037760*) and *MYRCENE SYNTHASE* (*LOC115716405*,* LOC133029092*,* LOC133037756*). Some of the most downregulated genes included *(E-E)-GERANYLLINALOOL SYNTHASE* (14.8-fold compared to uninfected plants at 7 dpi) and *ALPHA-HUMULENE SYNTHASE* (*LOC115725506;* 14.6-fold compared to uninfected plants at 5 dpi).

### Differential gene expression analysis of *S. sclerotiorum* infecting *C. sativa* identified biological processes associated with carbohydrate metabolic activity and redox processing

**Fig. 7:**
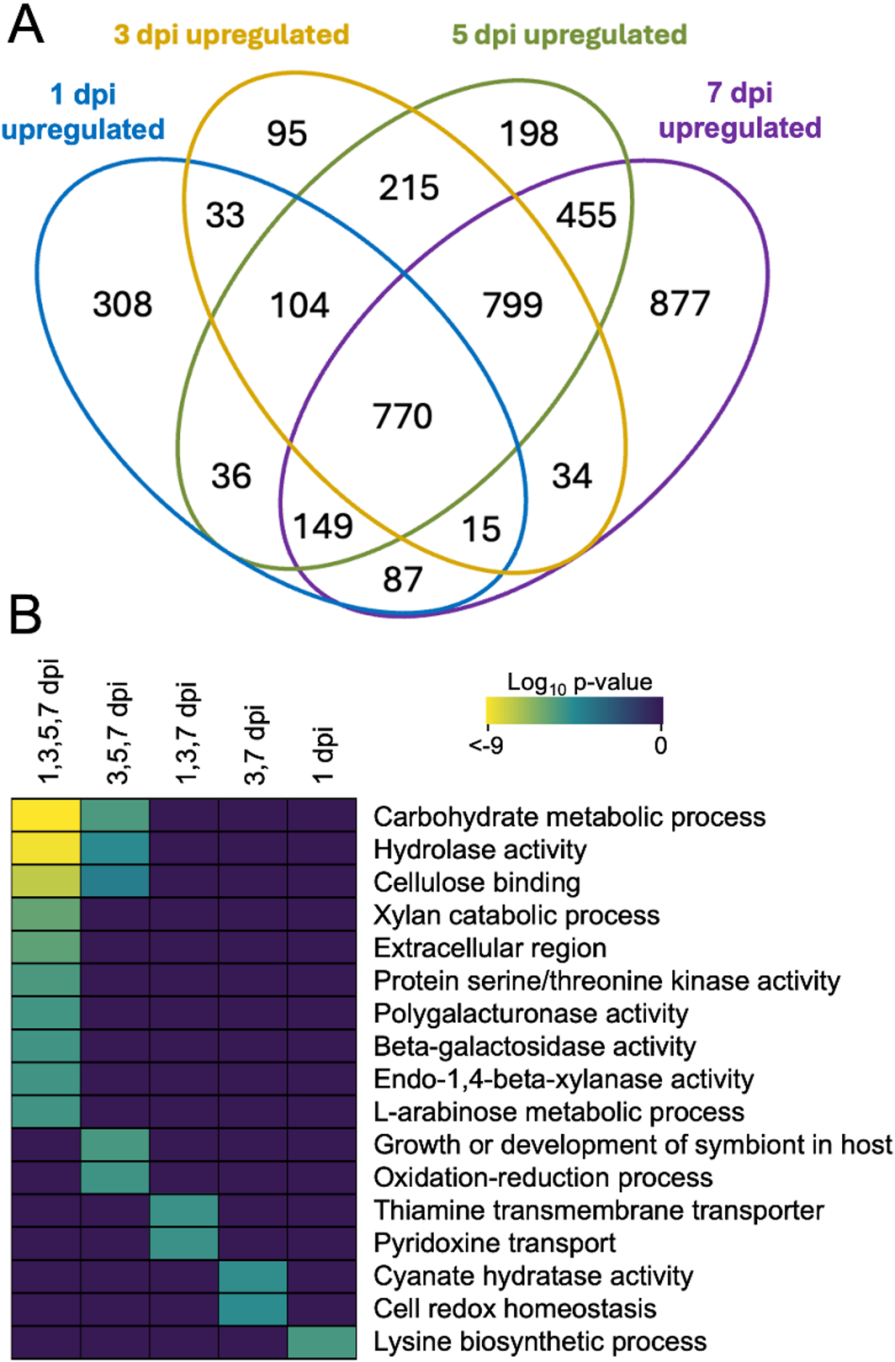
Upregulated differential gene expression of *S. sclerotiorum* infecting *C. sativa* across a seven-day infection period. (**A**) Venn diagram of significantly upregulated differentially expressed gene sets (FDR < 0.05) in response to infection. (**B**) Heatmap of significantly enriched GO terms (FDR < 0.01) resulting from timepoint-specific and shared subsets. A brighter yellow colour indicates greater statistical significance. Differentially expressed genes were compared to in vitro grown *S. sclerotiorum*. dpi = days post inoculation.

To investigate changes in *S. sclerotiorum* gene activity during *C. sativa* infection, we carried out differential gene expression and gene ontology (GO) term enrichment analysis (Fig. 7). Differential expression analysis revealed a high degree of shared differentially expressed genes at all sample timepoints (770 upregulated DEGs; Fig. 7A). Further, we observed the specific upregulation of 308 DEGs at 1 dpi, 95 at 3 dpi, 198 at 5 dpi, and 877 at 7 dpi. GO terms associated with host plant cell wall breakdown and carbohydrate metabolism (carbohydrate metabolic process, cellulose binding, xylan catabolic process and polygalacturonase activity) in addition to protein serine/threonine kinase activity were shared across all time points. GO terms associated with carbohydrate and cell wall breakdown, together with fungal growth and development within the host were enriched in gene sets shared between 3-, 5-, and 7- dpi. Redox processes and homeostasis were enriched in both the 3-, 5-, 7 dpi shared group, and the 3-, 7 dpi shared group (Fig. 7B).

Differential gene expression analysis of downregulated genes identified large numbers of shared DEGs between all sampled time points (610 genes) and shared between 3-, 5- and 7 dpi (710 genes; Supplementary Figure S7). Gene ontology terms enriched across all sampled timepoints identified enriched GO terms associated with translation and protein folding (ribosome, translation, unfolded protein binding, and protein folding) in addition to mitochondrial activity (mitochondrion and tricarboxylic acid cycle).

### Validation of RNA Sequencing Results with RT-qPCR

To validate the findings of our RNA sequencing experiments, we studied the relative abundance of four *C. sativa* defense markers including *PR-1*,* PR-10a*,* PR-5*, and *ENDOCHITINASE-2* using reverse-transcriptase quantitative PCR (RT-qPCR). Data show similar levels of gene activity of the four selected transcripts in both the RNA sequencing counts data and relative mRNA abundance observed using RT-qPCR (Figure S8).

## Discussion

Known as both a stem and bud rot pathogen, *S. sclerotiorum* is regarded as an emerging fungal pathogen of *C. sativa*^19^. This study serves as the first description of the *Cannabis-Sclerotinia* pathosystem at the mRNA level where we show the rapid initiation of infection in the *C. sativa* inflorescence. Within one week of inoculation, severe disease symptoms were widespread in the plant. Global gene activity underpinning this interaction revealed *C. sativa* responded to infection through the elicitation of lignin deposition, redox buffering and generalized plant defense/immune hormone signalling cascades. Anatomical investigation at the site of inoculation showed the rapid colonization and degradation of host plant tissues by *S. sclerotiorum* starting in the epidermis and mesophyll before targeting the vascular system of the plant.

Detection of plant pathogens ties together the concepts of PTI and ETI, where unique molecular responses are initiated resulting from the recognition of pathogenic elicitors, PAMPs or DAMPs^37,41^. In response to *S. sclerotiorum* infection, we uncovered the upregulation of genes whose products are involved in pathogen perception and early defense responses such as ser/thr RLKs, NLRs, wall associated kinases (WAK) and WAK-like proteins (WAKL)^41,42^. WAKs and WAKLs have long been known to play a role in the plant defense response through binding pectin and oligosaccharides which act as DAMPs during biotic stress responses^43–45^. Expression of WAKs have previously been seen to be upregulated in *Arabidopsis thaliana* in response to SA and wounding, and have been associated with resistance against both hemibiotrophic and necrotrophic pathogens through pathogen- or host-derived elicitor detection, and subsequent cell wall restructuring^44,46^. In addition to *A. thaliana*, immunity-related WAKs/WAKLs have been documented in various crop species that include *Triticum aestivum* (wheat), *Oryza sativa* (rice), *Hordeum vulgare* (barley), *Zea mays* (maize), *Sesamum indicum* (sesame), *Solanum lycopersicum* (tomato), *Gossypium hirsutum* (cotton), and *Brassica napus* (canola) thereby suggesting WAKs/WAKLs as an evolutionarily conserved feature of the plant defense response against fungal necrotrophs^44,45,47^. Notable upregulation of *C. sativa WAK2* and *WAKL1* midway through the seven-day infection period begs the questions of whether earlier induction of these genes would result in greater host resistance, leaving room for further study.

The plant cell wall is the first line of defencse serving as a barrier to restrict attacking pathogens. Fungal necrotrophic pathogens make use of CWDEs to impair cell wall integrity and ultimately degrade host plant tissues^48^. Despite the presence of *S. sclerotiorum* throughout the epidermis, mesophyll and vasculature of the *C. sativa* leaf at 3 dpi, cell wall degradation at this timepoint was not yet observed. In comparison, previous studies have detailed extensive cellular degradation resulting from *S. sclerotiorum* infection as early as 2 dpi in leaves of *B. napus*^28^ and *A. thaliana*^33^, and 3 dpi in *Glycine max*^49^(soybean). Our data show that *S. sclerotiorum* progressed into *C. sativa* leaf tissues more slowly, however it should be noted that this delay could be the result of the complex three-dimensional structure of the cola, versus the direct inoculation of the leaf as was used in the above studies. Work carried out by Wytinck et al. (2022) in the *B. napus* stem showed similar findings where *S. sclerotiorum* infection resulted in extensive colonization and degradation of the host epidermis, cortex, and phloem, while xylem tissues remained largely intact. These results suggest a common infection strategy by *S. sclerotiorum* across diverse plant species, despite the structural and complex metabolic differences underpinning *C. sativa*.

Our GO enrichment analysis revealed induction of cell wall degrading activities in *S. sclerotiorum* as infection was initiated. Hydrolase activity, xylan catabolic process, polygalacturonase activity, beta-galactosidase activity, and 1,4-beta-xylanase activity were found across all infection time points. Production of CWDEs by *S. sclerotiorum* facilitates tissue penetration and maceration, through cell wall weakening characteristic of necrotrophic fungal infection^22^. Polygalacturonases (PGs) are a class of fungal pectinases that target unesterified pectate polymers of the middle lamella and primary cell wall of the host plant^22^. As plant cell walls are weakened and degraded by PGs, the secreted oligogalacturonides have been shown to elicit ROS burst, including H_2_O_2_ and O_2_^−^, as the plant attempts to restrict pathogen attack^22,50^. ROS production to develop localized cell death occurring as a result of pathogen infection is termed as the plant hypersensitive response (HR), and is regarded as one of the most important factors in impeding the growth of biotrophic pathogens^51^. Although the HR is generally effective against biotrophs, necrotrophic pathogen virulence, including that of *S. sclerotiorum* and *B. cinerea*, has been suggested to be strengthened as a result of HR elicitation^29,52^. Studies in *Nicotiana tabacum* (tobacco) and *A. thaliana* have also revealed that plants unable to initiate the HR demonstrated increased resistance to *S. sclerotiorum*^53,54^. While we uncovered rapid upregulation of *S. sclerotiorum* PG activity that continued throughout the seven-day infection period, the *C. sativa* HR was not induced until 3 dpi, likely revealing one facet of the *S. sclerotiorum* coordinated and timed control of host ROS activation to favour fungal proliferation in host tissues.

Similarly, the major *S. sclerotiorum* pathogenicity factor oxalic acid, has also been previously found to manipulate host ROS production to initially suppresses ROS signalling, before eliciting ROS production, leading to cell death^28,55^. Recent studies have challenged the classical view of the *S. sclerotiorum* necrotrophic lifestyle, suggesting the possibility of a brief biotrophic phase early in the infection process. These studies suggest that *S. sclerotiorum* is capable of suppressing SA-mediated SAR early in infection, and thus the HR, before the true necrotrophic portion of the lifestyle occurs in which initiation of ROS production leads to localized cell death and subsequent widespread infection^56,57^. Our results support the initial suppression of SA-dependent SAR as *PHENYLALANINE AMMONIA-LYASE* (*PAL*), a major enzyme involved in SA biosynthesis, was not upregulated until 3 dpi, with highest upregulation occurring two days later, at 5 dpi. Similarly, we found *GLUTATHIONE S-TRANSFERASE U10* and *GLUTATHIONE S-TRANSFERASE* were not upregulated until 3 dpi. Glutathione transferases are cellular protectant enzymes involved in redox homeostasis and ROS detoxification that are rapidly induced by H_2_O_2_^58^. Previous work in *B. napus* revealed plants partially resistant to *S. sclerotiorum* demonstrated increased redox buffering capacity as early as 1 dpi^28^. These results suggest that a more rapid induction of redox buffering by *C. sativa* may result in an increased resistance or tolerant phenotype.

In addition to ROS accumulation, *S. sclerotiorum* infection has previously been found to initiate lignin biosynthesis within the host plant^59^. Lignins are biopolymers important for plant cell wall structural support, and have previously been reported to be deposited in *Brassica* species in response to *S. sclerotiorum* infection^60^. Specific to our study, we found that in response to *S. sclerotiorum* infection, *C. sativa* highly upregulated *PAL* in addition to various class III peroxidases that include *PEROXIDASE 57*,* CATIONIC PEROXIDASE 1*,* LIGNIN-FORMING ANIONIC PEROXIDASE*, and *PEROXIDASE 5-LIKE*. Belonging to the PR-9 subfamily of PR proteins, class III plant peroxidase gene expression has previously been found to increase in plants challenged with fungi, in addition to bacteria, viruses and viroids^61–63^. Plant peroxidases are also capable of creating physical barriers to limit pathogen invasion in response to stimuli such as wounding, pathogen presence, or hormone accumulation^61^. The upregulation of peroxidase activity uncovered in this study serves as the first description of genes involved in H_2_O_2_-dependent lignin deposition by *C. sativa* to restrict further incursion of *S. sclerotiorum*.

While PAL is known to be involved in lignification, it is also involved in SA biosynthesis^64,65^. SA biosynthesis is initiated during both ETI and PTI in response to recognition of PAMPs or pathogenic effectors^66^. Increased SA levels are required for plant SAR initiation, which is accompanied and characterized by increased systemic PR gene expression^39^. Belonging to the PR families PR-3, -4, -8, and − 11, chitinases are among the most abundant PR proteins^67^. Our dataset revealed the upregulation of chitinase genes across the *S. sclerotiorum* infection process. Additionally, we also observed increases in gene activity of the PR-5 and PR-10 subfamilies. It is thought that PR-5 proteins exhibit antifungal activity by inserting themselves into fungal membranes to create a transmembrane pore, later leading to influx of water and subsequent fungal osmotic rupture^68^. Unlike the PR-5 proteins however, the function of PR-10 proteins remains largely unclear, which may be attributed to the large multi-gene families they code for^68,69^. As our study serves as one of the first transcriptome-level investigations of the infection of *C. sativa* with any fungal pathogen, future studies that explore PR protein activity in other fungal interactions with *C. sativa* may reveal how PR gene expression may be engineered or selected to develop more resistant germplasm.

As complex specialized secondary plant metabolites, terpenes serve various purposes to plants that include attracting pollinators and insect predators of feeding herbivores, and creating both chemical and physical barriers to herbivorous insects, as well as invading pathogens^11,12^. Specific to our study, we uncovered differential expression activity of various *C. sativa* terpene synthases. While terpene synthase activity of *C. sativa* in response to biotic stressors has not previously been investigated, our results suggest that the largescale transcriptional reprogramming that occurs as a result of coordinating a defense response against *S. sclerotiorum* may impact the terpenoid profile exhibited by infected plants. As the terpene composition of the resin produced by glandular trichomes of the female inflorescence is largely responsible for the scent and flavour characteristics of harvested *Cannabis* products, terpenes greatly impact consumer preferences^6^. Consequently, terpenoid profiles often serve as a basis for modern selective breeding. With varying terpenoid profiles attributed to unique *C. sativa* cultivars, the question arises of how different cultivars may respond to pathogen attack based on terpenoid profile composition; and whether selective breeding may allow for the production of cultivars that exhibit an increased resistance phenotype.

Taken together, this study provides a comprehensive investigation into the transcriptional and anatomical changes that occur as *S. sclerotiorum* initiates infection in the *C. sativa* inflorescence. Our data reveal large shifts in host gene activity in response to infection with *S. sclerotiorum* that largely peak at 5 dpi. Gene categories identified in *C. sativa* show complex shifts in defense hormone signalling and redox buffering associated with the plant immune responses across time post inoculation. Anatomical study revealed extensive degradation of host cortical and vascular phloem tissues associated with the production of fungal toxins and CWDEs. Additional studies conducting molecular and biochemical validation of the various gene products and metabolites identified herein will allow for increased understanding of this pathosystem and can help to direct future crop improvement studies.

## Materials and methods

### *Cannabis sativa* growth conditions

Female *C. sativa* plants, cultivar ‘Kona’, were sourced from Rogue Botanical, a licensed grower in southern Manitoba, Canada. Plants were obtained in vegetative growth, 20 days after being clonally propagated. At 22 days old, plants were transplanted from 4-inch pots to 6-inch pots in Sunshine growing mix #4 (Sungro, Agawan, MA, USA). Plants were grown in a controlled environment chamber under long day conditions (18 h light, 6 h dark), 500 µmol/m^2^/s^− 1^, 23 °C and 50% relative humidity. After 30 days in vegetative growth, the photoperiod was adjusted to 12 h light, 12 h dark to promote flowering. Plants were fertilized using Advanced Nutrients Sensi Grow/Bloom nutrient packages, as per manufacturer’s instructions (Advanced Nutrients, West Hollywood, CA, USA).

Experimental research on *C. sativa*, including the collection of plant material, were conducted in accordance with relevant institutional, national, and international guidelines and legislation.

### Sclerotinia sclerotiorum inoculation of the Cannabis sativa cola

*S. sclerotiorum* was grown in vitro on potato dextrose agar (BD Difco) plates supplemented with 15 µg/mL tetracycline HCl. *S. sclerotiorum* mycelial plugs were taken from the leading edge of a 3-day-old actively growing plate using a P1000 pipette tip. Mycelial plugs were carefully placed at the inflorescence node of the third-most distal inflorescence of the *C. sativa* cola using forceps. Infection took place over a seven-day period with colas being harvested after 1-, 3-, 5- and 7- days post inoculation (dpi). Both infected and untreated control (UTC) colas were harvested at each timepoint. Twelve *C. sativa* plants were used for this experiment with four biological replicates sampled across each timepoint.

### Sample collection, RNA isolation, library preparation and RNA sequencing

Harvested colas were immediately trimmed down to the main floral stem, while maintaining ~ 1 cm^3^ of floral tissue of the inoculated inflorescence and immediately flash frozen using liquid nitrogen. Tissue was ground to a fine powder using a mortar and pestle with liquid nitrogen prior to RNA extraction.

RNA was extracted using the Purelink Plant RNA Reagent (Invitrogen, Waltham, MA, USA) as per manufacturer’s protocol. Following RNA extraction, Qiagen’s RNeasy Plant Minikit and RNase-Free DNase Set was used for DNAse treatment following the “RNA Cleanup” protocol available in Qiagen’s RNeasy Mini Handbook (Qiagen, Toronto, ON, Canada). As sample purity was often compromised as a result of the DNAse treatment procedure, samples then underwent a sodium acetate precipitation. This precipitation used 3M C_2_H_3_NaO_2_ (pH 5.2) and subsequent ethanol washes (100% followed by 75%) before resuspension in molecular grade water to yield RNA of increased purity.

cDNA libraries were constructed by Genome Québec following their polyA Enriched RNA Library Preparation protocol. Paired-end 100 bp reads were sequenced for a minimum of 25 million reads per library on the Illumina NovaSeq sequencing system at Genome Québec (Montréal, Québec, Canada). All sequencing data can be found at the Gene Expression Omnibus, under accession GSE284432.

### Fungal load qPCR and RT-qPCR

Expression of *PATHOGENESIS RELATED PROTEIN 1* (*PR-1; LOC115704466*), *PATHOGENESIS-RELATED PROTEIN STH-2* (*PR-10a; LOC115722015*), *THAUMATIN-LIKE PROTEIN 1B* (*PR-5; LOC115710654*), and *ENDOCHITINASE 2* (*LOC115705823*) were assessed using RT-qPCR. cDNA was synthesized using qScript™ cDNA SuperMix according to manufacturer’s instructions (Quantabio, Beverly, MA, USA). To quantify defense gene transcript abundance, RT-qPCR was run on *PR1*,* PR-10a*,* PR-5*,* and ENDOCHITINASE 2* with the housekeeping genes *TIP41-LIKE PROTEIN* (*TIP41*; *LOC115703022*) and *ADENINE PHOSPHO-RIOSYLTRANSFERASE 1* (*APT1*; *LOC115713640*) used as internal controls^70^. Primer sequence information is found in Table S1.

We also used qPCR to determine the relative fungal load between samples. Genomic DNA was extracted from ground tissue using a modified cetyltrimethylammonium bromide (CTAB) method^71^. As a target for *S. sclerotiorum*, we used 18 S rDNA as described in Wytinck et al. (2022). Primer sequences and target loci for both RT-qPCR and fungal load qPCR can be found in Supplementary Table S1. SsoFast EvaGreen Supermix was used as per manufacturer’s instructions for both RT-qPCR and qPCR (Bio-Rad Laboratories, Hercules, CA, USA).

### RNA sequencing analysis

Raw reads were processed using computing clusters available through Compute Canada and the Digital Research Alliance of Canada (https://www.alliancecan.ca/en). Prior to read alignment, sequence read quality was first assessed using FastQC (v0.12.1; https://www.bioinformatics.babraham.ac.uk/projects/fastqc/)^72^. Paired-end read alignment was carried out using the *C. sativa* cultivar ‘Pink Pepper’ reference genome (NCBI RefSeq assembly GCF_029168945.1) and the *S. sclerotiorum* reference genome^73,74^ (NCBI RefSeq assembly GCF_000146945.2; ) using HISAT2 (v2.2.1; https://daehwankimlab.github.io/hisat2/)^75^. Transcript abundance was determined using featureCounts (v2.0.3; https://subread.sourceforge.net/)^76^. With one of the barriers to working with the *C. sativa* transcriptome being the level of genome annotation, we used predicted protein orthologs publicly available for the loci of the *C. sativa* cultivar ‘Pink Pepper’ genome through the NCBI Genomes database (RefSeq accession GCF_029168945.1; Lim, 2023). Differential gene expression analysis, low counts filtering, library normalization, principle component analysis and further data visualization was done using libraries DESeq2 (v1.42.1; https://bioconductor.org/packages/release/bioc/html/DESeq2.html)^77^, ashr (v2.2.63; https://cran.r-project.org/web/packages/ashr/index.html)^78^, and ggplot2 (v3.5.2; https://cran.r-project.org/web/packages/ggplot2/index.html)^79^ in R (v4.3.1; https://www.r-project.org/)^80^. Genes with counts lower than 10 across all samples were filtered prior to normalization and differential gene expression analysis. Raw sequenced read counts were normalized using the median of ratios method in DESeq2^81^. Differentially expressed genes were called with a p-value < 0.01 when adjusted for false discovery rate (FDR) by the Benjamini-Hochberg method^82^. GO term enrichment was carried out on differentially expressed gene sets using SeqEnrich (v2.0)^83,84^. GO and DEG lists (Supplementary Material 1), raw counts aligned to *C. sativa* (Supplementary Material 3), and raw counts aligned to *S. sclerotiorum* (Supplementary Material 4) are provided as supplemental datasets.

### Sample preparation for light microscopy

Sample preparation, sectioning and staining followed the methods previously described by Chan and Belmonte (2013) with slight modifications. Harvested colas were trimmed to the above-mentioned region of interest before being fixed in a solution of 2.5% glutaraldehyde and 1.6% paraformaldehyde in 1x phosphate-buffered saline. Tissue was added to fixative solution before being vacuum infiltrated for 30 min to ensure adequate penetration of the fixative into the *C. sativa* tissues. Tissue samples were fixed for 24 h at 4 °C. Tissue was decoloured in methyl cellosolve for 24 h, followed by daily 100% ethanol changes for three days at 4 °C. Historesin (Leica Microsystems, Wetzlar, Germany) was gradually infiltrated into processed tissue using a 30%, 50%, 75% and 100% ethanol: historesin mixture. Pure Historesin was exchanged three times over the course of a week, while vacuum infiltrating the tissue in for 30 min halfway through this period. Tissue was then embedded in round molds using an embedding medium composed of 91.5% Historesin, 2.4% polyethylene glycol 400, and 6.1% Historesin Hardener (Leica Microsystems, Wetzlar, Germany; Chan & Belmonte, 2013).

### Sectioning and staining for light microscopy

Hardened Historesin blocks were sectioned at 3 μm using disposable Epredia Edge-Rite steel blades (Epredia, Kalamazoo, MI, USA) mounted on a Leica RM2245 microtome (Leica Microsystems, Wetzlar, Germany). Sections were placed on glass slides for staining.

Sections were first stained with periodic acid-Schiff stain (15 min in 0.1% periodic acid, followed by 15 min in Schiff’s reagent) before being stained with 0.1% toluidine blue O suspended in distilled water for 30 s. Following staining, coverslips were mounted on the slides using Cytoseal 60 (Richard-Allen Scientific, Kalamazoo, MI, USA). Slides were viewed using a brightfield light microscope and micrographs were taken using the Leica Application Suite software version 4.6.0 (Leica Microsystems, Wetzlar, Germany). Image cropping and the addition of scale bars was carried out in Adobe Photoshop version 25.7.0 (Adobe Systems Inc., San Jose, CA, USA).

## Supplementary Information

Below is the link to the electronic supplementary material.

Supplementary Material 1

Supplementary Material 2

Supplementary Material 3

Supplementary Material 4

## Data Availability

All RNA sequencing data is publicly available online via the Gene Expression Omnibus at the accession GSE284432.

## References

[1] Long, T., Wagner, M., Demske, D., Leipe, C. & Tarasov, P. E. Cannabis in Eurasia: origin of human use and Bronze Age trans-continental connections. *Veg. Hist. Archaeobotany*. **26**, 245–258 (2017).

[2] Ren, G. et al. Large-scale whole-genome resequencing unravels the domestication history of Cannabis sativa. *Sci. Adv.* **7**, eabg2286 (2021). 34272249 10.1126/sciadv.abg2286 PMC8284894

[3] Braich, S., Baillie, R. C., Jewell, L. S., Spangenberg, G. C. & Cogan, N. O. I. Generation of a Comprehensive Transcriptome Atlas and Transcriptome Dynamics in Medicinal Cannabis. *Sci. Rep.* **9**, 16583 (2019). 31719627 10.1038/s41598-019-53023-6 PMC6851104

[4] van Bakel, H. et al. The draft genome and transcriptome of Cannabis sativa. *Genome Biol.* **12**, R102 (2011). 22014239 10.1186/gb-2011-12-10-r102 PMC3359589

[5] Divashuk, M. G., Alexandrov, O. S., Razumova, O. V., Kirov, I. V. & Karlov, G. I. Molecular Cytogenetic Characterization of the Dioecious Cannabis sativa with an XY Chromosome Sex Determination System. *PLOS ONE*. **9**, e85118 (2014). 24465491 10.1371/journal.pone.0085118 PMC3897423

[6] Booth, J. K., Page, J. E. & Bohlmann, J. Terpene synthases from Cannabis sativa. *PLoS ONE*. **12**, e0173911 (2017). 28355238 10.1371/journal.pone.0173911 PMC5371325

[7] Rodziewicz, P., Loroch, S., Marczak, Ł., Sickmann, A. & Kayser, O. Cannabinoid synthases and osmoprotective metabolites accumulate in the exudates of *Cannabis sativa* L. glandular trichomes. *Plant. Sci.* **284**, 108–116 (2019). 31084863 10.1016/j.plantsci.2019.04.008

[8] Xie, Z. et al. Cannabis sativa: origin and history, glandular trichome development, and cannabinoid biosynthesis. *Hortic. Res.* **10**, uhad150 (2023). 37691962 10.1093/hr/uhad150 PMC10485653

[9] Booth, J. K. & Bohlmann, J. Terpenes in *Cannabis sativa* – From plant genome to humans. *Plant. Sci.* **284**, 67–72 (2019). 31084880 10.1016/j.plantsci.2019.03.022

[10] Fischedick, J. T. Identification of Terpenoid Chemotypes Among High (–)-trans-∆9- Tetrahydrocannabinol-Producing Cannabis sativa L. Cultivars. *Cannabis Cannabinoid Res.* **2**, 34–47 (2017). 28861503 10.1089/can.2016.0040 PMC5436332

[11] Chen, F., Tholl, D., Bohlmann, J. & Pichersky, E. The family of terpene synthases in plants: a mid-size family of genes for specialized metabolism that is highly diversified throughout the kingdom. *Plant. J.* **66**, 212–229 (2011). 21443633 10.1111/j.1365-313X.2011.04520.x

[12] Keeling, C. I. & Bohlmann, J. Genes, enzymes and chemicals of terpenoid diversity in the constitutive and induced defence of conifers against insects and pathogens. *New. Phytol*. **170**, 657–675 (2006). 16684230 10.1111/j.1469-8137.2006.01716.x

[13] Government of Canada. Consolidated federal laws of Canada, Cannabis Regulations. (2018). https://laws-lois.justice.gc.ca/eng/regulations/SOR-2018-144/FullText.html

[14] Hammond, D. et al. Evaluating the impacts of cannabis legalization: The International Cannabis Policy Study. *Int. J. Drug Policy*. **77**, 102698 (2020). 32113149 10.1016/j.drugpo.2020.102698

[15] Government of Canada. Licensed area market data. (2024). https://www.canada.ca/en/health-canada/services/drugs-medication/cannabis/research-data/market/licensed-area.html

[16] Bains, P. S., Bennypaul, H. S., Blade, S. F. & Weeks, C. First Report of Hemp Canker Caused by Sclerotinia sclerotiorum in Alberta, Canada. *Plant. Dis.* **84**, 372–372 (2000). 30841263 10.1094/PDIS.2000.84.3.372B

[17] Garfinkel, A. R. First Report of Sclerotinia sclerotiorum Causing Stem Canker on Cannabis sativa in Oregon. *Plant. Dis.* **105**, 2245 (2021).

[18] Punja, Z. K. Emerging diseases of Cannabis sativa and sustainable management. *Pest Manag Sci.* **77**, 3857–3870 (2021). 33527549 10.1002/ps.6307 PMC8451794

[19] Punja, Z. K. & Ni, L. The bud rot pathogens infecting cannabis (Cannabis sativa L., marijuana) inflorescences: symptomology, species identification, pathogenicity and biological control. *Can. J. Plant. Pathol.* **43**, 827–854 (2021).

[20] Thiessen, L. D., Schappe, T., Cochran, S., Hicks, K. & Post, A. R. Surveying for Potential Diseases and Abiotic Disorders of Industrial Hemp (Cannabis sativa) Production. *Plant. Health Prog*. **21**, 321–332 (2020).

[21] Yang, X., Justice, A. & Colburn, C. First report of Sclerotinia sclerotiorum causing stem canker on industrial hemp in South Carolina, USA. *Plant. Dis.*10.1094/PDIS-04-23-0700-PDN (2023).37707825

[22] Bolton, M. D., Thomma, B. P. H. J. & Nelson, B. D. Sclerotinia sclerotiorum (Lib.) de Bary: biology and molecular traits of a cosmopolitan pathogen. *Mol. Plant. Pathol.* **7**, 1–16 (2006). 20507424 10.1111/j.1364-3703.2005.00316.x

[23] Liang, X. & Rollins, J. A. Mechanisms of Broad Host Range Necrotrophic Pathogenesis in Sclerotinia sclerotiorum. Phytopathology^®^**108**, 1128–1140 (2018). 30048598 10.1094/PHYTO-06-18-0197-RVW

[24] Saharan, G. S. & Mehta, N. *Sclerotinia Diseases of Crop Plants: Biology, Ecology and Disease Management* (Springer Science & Business Media, 2008).

[25] Willbur, J., McCaghey, M., Kabbage, M. & Smith, D. L. An overview of the Sclerotinia sclerotiorum pathosystem in soybean: impact, fungal biology, and current management strategies. *Trop. Plant. Pathol.* **44**, 3–11 (2019).

[26] Alkooranee, J. T. et al. Detecting the Hormonal Pathways in Oilseed Rape behind Induced Systemic Resistance by Trichoderma harzianum TH12 to Sclerotinia sclerotiorum. *PLOS ONE*. **12**, e0168850 (2017). 28045929 10.1371/journal.pone.0168850 PMC5207704

[27] Hossain, M. M., Sultana, F., Li, W., Tran, L. S. P. & Mostofa, M. G. Sclerotinia sclerotiorum (Lib.) de Bary: Insights into the Pathogenomic Features of a Global Pathogen. *Cells* **12**, 1063 (2023). 37048136 10.3390/cells12071063 PMC10093061

[28] Girard, I. J. et al. RNA sequencing of Brassica napus reveals cellular redox control of Sclerotinia infection. *J. Exp. Bot.* **68**, 5079–5091 (2017). 29036633 10.1093/jxb/erx338 PMC5853404

[29] Hegedus, D. D. & Rimmer, S. R. Sclerotinia sclerotiorum: When to be or not to be a pathogen? *FEMS Microbiol. Lett.* **251**, 177–184 (2005). 16112822 10.1016/j.femsle.2005.07.040

[30] Horbach, R., Navarro-Quesada, A. R., Knogge, W. & Deising, H. B. When and how to kill a plant cell: Infection strategies of plant pathogenic fungi. *J. Plant. Physiol.* **168**, 51–62 (2011). 20674079 10.1016/j.jplph.2010.06.014

[31] Derbyshire, M. C. & Denton-Giles, M. The control of sclerotinia stem rot on oilseed rape (Brassica napus): current practices and future opportunities. *Plant. Pathol.* **65**, 859–877 (2016).

[32] Wytinck, N. et al. Host induced gene silencing of the Sclerotinia sclerotiorum ABHYDROLASE-3 gene reduces disease severity in Brassica napus. *PLOS ONE*. **17**, e0261102 (2022). 36018839 10.1371/journal.pone.0261102 PMC9417021

[33] Walker, P. L. et al. Control of white mold (Sclerotinia sclerotiorum) through plant-mediated RNA interference. *Sci. Rep.* **13**, 6477 (2023). 37081036 10.1038/s41598-023-33335-4 PMC10119085

[34] Ghozlan, M. H., EL-Argawy, E., Tokgöz, S., Lakshman, D. K. & Mitra, A. Plant Defense against Necrotrophic Pathogens. *Am. J. Plant. Sci.* **11**, 2122–2138 (2020).

[35] Song, W., Forderer, A., Yu, D. & Chai, J. Structural biology of plant defence. *New. Phytol*. **229**, 692–711 (2021). 32880948 10.1111/nph.16906

[36] Walker, P. L. et al. Tissue-specific mRNA profiling of the Brassica napus–Sclerotinia sclerotiorum interaction uncovers novel regulators of plant immunity. *J. Exp. Bot.* **73**, 6697–6710 (2022). 35961003 10.1093/jxb/erac333

[37] Pruitt, R. N., Gust, A. A. & Nürnberger, T. Plant immunity unified. *Nat. Plants*. **7**, 382–383 (2021). 33785867 10.1038/s41477-021-00903-3

[38] Cale, N. L. et al. Global mRNA profiling reveals the effect of boron as a crop protection tool against Sclerotinia sclerotiorum. *AoB PLANTS*. **plae056**10.1093/aobpla/plae056 (2024). 10.1093/aobpla/plae056 PMC11551614 39529684

[39] Pieterse, C. M. J. et al. Induced Systemic Resistance by Beneficial Microbes. *Annu. Rev. Phytopathol.* **52**, 347–375 (2014). 24906124 10.1146/annurev-phyto-082712-102340

[40] Yu, Y. et al. Induced Systemic Resistance for Improving Plant Immunity by Beneficial Microbes. *Plants* **11**, 386 (2022). 35161366 10.3390/plants11030386 PMC8839143

[41] Dodds, P. N., Chen, J. & Outram, M. A. Pathogen perception and signaling in plant immunity. *Plant. Cell.* **koae020**10.1093/plcell/koae020 (2024). 10.1093/plcell/koae020 PMC11062475 38262477

[42] Kohorn, B. D. & Kohorn, S. L. The cell wall-associated kinases, WAKs, as pectin receptors. *Front Plant. Sci.* **3**, 88 (2012). 10.3389/fpls.2012.00088 PMC3355716 22639672

[43] Harvey, A., van den Berg, N. & Swart, V. Describing and characterizing the WAK/WAKL gene family across plant species: a systematic review. *Front Plant. Sci.* **15**, 1467148 (2024). 10.3389/fpls.2024.1467148 PMC11588464 39600901

[44] Stephens, C., Hammond-Kosack, K. E. & Kanyuka, K. WAKsing plant immunity, waning diseases. *J. Exp. Bot.* **73**, 22–37 (2022). 34520537 10.1093/jxb/erab422

[45] Yan, W. et al. Genome-wide characterization of the wall-associated kinase-like (WAKL) family in sesame (Sesamum indicum) identifies a SiWAKL6 gene involved in resistance to Macrophomina Phaseolina. *BMC Plant. Biol.* **23**, 624 (2023). 38057720 10.1186/s12870-023-04658-1 PMC10702004

[46] He, Z. H., He, D. & Kohorn, B. D. Requirement for the induced expression of a cell wall associated receptor kinase for survival during the pathogen response. *Plant. J.* **14**, 55–63 (1998). 9681026 10.1046/j.1365-313x.1998.00092.x

[47] Wu, J. et al. Comparative transcriptomic analysis uncovers the complex genetic network for resistance to Sclerotinia sclerotiorum in Brassica napus. *Sci. Rep.* **6**, 19007 (2016). 26743436 10.1038/srep19007 PMC4705546

[48] Bellincampi, D., Cervone, F. & Lionetti, V. Plant cell wall dynamics and wall-related susceptibility in plant–pathogen interactions. *Front Plant. Sci.* **5**, 228 (2014). 10.3389/fpls.2014.00228 PMC4036129 24904623

[49] Davidson, A. L. et al. Histopathology of Sclerotinia sclerotiorum infection and oxalic acid function in susceptible and resistant soybean. *Plant. Pathol.* **65**, 878–887 (2016).

[50] Wojtaszek, P. Oxidative burst: an early plant response to pathogen infection. *Biochem. J.* **322**, 681–692 (1997). 9148737 10.1042/bj3220681 PMC1218243

[51] Govrin, E. M. & Levine, A. The hypersensitive response facilitates plant infection by the necrotrophic pathogen Botrytis cinerea. *Curr. Biol.* **10**, 751–757 (2000). 10898976 10.1016/s0960-9822(00)00560-1

[52] Rossi, F. R. et al. Reactive oxygen species generated in chloroplasts contribute to tobacco leaf infection by the necrotrophic fungus Botrytis cinerea. *Plant. J.* **92**, 761–773 (2017). 28906064 10.1111/tpj.13718

[53] Dickman, M. B. et al. Abrogation of disease development in plants expressing animal antiapoptotic genes. *Proc. Natl. Acad. Sci.* 98, 6957–6962 (2001). 10.1073/pnas.091108998 PMC34460 11381106

[54] Thomma, B. P., Penninckx, I. A., Cammue, B. P. & Broekaert, W. F. The complexity of disease signaling in *Arabidopsis*. *Curr. Opin. Immunol.* **13**, 63–68 (2001). 11154919 10.1016/s0952-7915(00)00183-7

[55] Cessna, S. G., Sears, V. E., Dickman, M. B. & Low, P. S. Oxalic Acid, a Pathogenicity Factor for Sclerotinia sclerotiorum, Suppresses the Oxidative Burst of the Host Plant. *Plant. Cell.* **12**, 2191–2199 (2000). 11090218 10.1105/tpc.12.11.2191 PMC150167

[56] Kabbage, M., Yarden, O. & Dickman, M. B. Pathogenic attributes of *Sclerotinia sclerotiorum*: Switching from a biotrophic to necrotrophic lifestyle. *Plant. Sci.* **233**, 53–60 (2015). 25711813 10.1016/j.plantsci.2014.12.018

[57] Seifbarghi, S. et al. Changes in the Sclerotinia sclerotiorum transcriptome during infection of Brassica napus. *BMC Genom.* **18**, 266 (2017). 10.1186/s12864-017-3642-5 PMC5372324 28356071

[58] Lamb, C. & Dixon, R. A. THE OXIDATIVE BURST IN PLANT DISEASE RESISTANCE. *Annu. Rev. Plant. Biol.* **48**, 251–275 (1997). 10.1146/annurev.arplant.48.1.251 15012264

[59] Calla, B. et al. Genomic evaluation of oxalate-degrading transgenic soybean in response to Sclerotinia sclerotiorum infection. *Mol. Plant. Pathol.* **15**, 563–575 (2014). 24382019 10.1111/mpp.12115 PMC6638623

[60] Uloth, M. B., Clode, P. L., You, M. P. & Barbetti, M. J. Attack modes and defence reactions in pathosystems involving Sclerotinia sclerotiorum, Brassica carinata, B. juncea and B. napus. *Ann. Bot.* **117**, 79–95 (2016). 26420204 10.1093/aob/mcv150 PMC4701150

[61] Almagro, L. et al. Class III peroxidases in plant defence reactions. *J. Exp. Bot.* **60**, 377–390 (2009). 19073963 10.1093/jxb/ern277

[62] van Loon, L. C., Rep, M. & Pieterse, C. M. J. Significance of Inducible Defense-related Proteins in Infected Plants. *Annu. Rev. Phytopathol.* **44**, 135–162 (2006). 16602946 10.1146/annurev.phyto.44.070505.143425

[63] Sasaki, K. et al. Ten Rice Peroxidases Redundantly Respond to Multiple Stresses Including Infection with Rice Blast Fungus. *Plant. Cell. Physiol.* **45**, 1442–1452 (2004). 15564528 10.1093/pcp/pch165

[64] Kim, D. S. & Hwang, B. K. An important role of the pepper phenylalanine ammonia-lyase gene (PAL1) in salicylic acid-dependent signalling of the defence response to microbial pathogens. *J. Exp. Bot.* **65**, 2295–2306 (2014). 24642849 10.1093/jxb/eru109 PMC4036500

[65] Ullah, C., Chen, Y. H., Ortega, M. A. & Tsai, C. J. The diversity of salicylic acid biosynthesis and defense signaling in plants: Knowledge gaps and future opportunities. *Curr. Opin. Plant. Biol.* **72**, 102349 (2023). 36842224 10.1016/j.pbi.2023.102349

[66] Mishina, T. E. & Zeier, J. Pathogen-associated molecular pattern recognition rather than development of tissue necrosis contributes to bacterial induction of systemic acquired resistance in Arabidopsis. *Plant. J.* **50**, 500–513 (2007). 17419843 10.1111/j.1365-313X.2007.03067.x

[67] Chouhan, R., Ahmed, S. & Gandhi, S. G. Over-expression of PR proteins with chitinase activity in transgenic plants for alleviation of fungal pathogenesis. *J. Plant. Pathol.* **105**, 69–81 (2023).

[68] Sinha, M. et al. Current Overview of Allergens of Plant Pathogenesis Related Protein Families. *Sci. World J.* 543195 (2014). (2014). 10.1155/2014/543195 PMC3947804 24696647

[69] Fernandes, H., Michalska, K., Sikorski, M. & Jaskolski, M. Structural and functional aspects of PR-10 proteins. *FEBS J.* **280**, 1169–1199 (2013). 23289796 10.1111/febs.12114

[70] Balthazar, C., Cantin, G., Novinscak, A., Joly, D. L. & Filion, M. Expression of Putative Defense Responses in Cannabis Primed by Pseudomonas and/or Bacillus Strains and Infected by Botrytis cinerea. *Front Plant. Sci.* **11**, 572112 (2020). 10.3389/fpls.2020.572112 PMC7723895 33324431

[71] Porebski, S., Bailey, L. G. & Baum, B. R. Modification of a CTAB DNA extraction protocol for plants containing high polysaccharide and polyphenol components. *Plant. Mol. Biol. Rep.* **15**, 8–15 (1997).

[72] Andrews, S. & FastQC A Quality Control tool for High Throughput Sequence Data. (2010). https://www.bioinformatics.babraham.ac.uk/projects/fastqc/

[73] Lim, J. D. Cannabis sativa Isolate KNU-18-1 (Cultivar: Pink Pepper), Whole Genome Sequencing Project. Genbank (2023).

[74] Amselem, J. et al. Genomic Analysis of the Necrotrophic Fungal Pathogens Sclerotinia sclerotiorum and Botrytis cinerea. *PLOS Genet.* **7**, e1002230 (2011). 21876677 10.1371/journal.pgen.1002230 PMC3158057

[75] Kim, D., Paggi, J. M., Park, C., Bennett, C. & Salzberg, S. L. Graph-based genome alignment and genotyping with HISAT2 and HISAT-genotype. *Nat. Biotechnol.* **37**, 907–915 (2019). 31375807 10.1038/s41587-019-0201-4 PMC7605509

[76] Liao, Y., Smyth, G. K. & Shi, W. featureCounts: an efficient general purpose program for assigning sequence reads to genomic features. *Bioinformatics* **30**, 923–930 (2014). 24227677 10.1093/bioinformatics/btt656

[77] Love, M. I., Huber, W. & Anders, S. Moderated estimation of fold change and dispersion for RNA-seq data with DESeq2. *Genome Biol.* **15**, 550 (2014). 25516281 10.1186/s13059-014-0550-8 PMC4302049

[78] Stephens, M. False discovery rates: a new deal. *Biostatistics* **18**, 275–294 (2017). 27756721 10.1093/biostatistics/kxw041 PMC5379932

[79] Wickham, H. ggplot2. *WIREs Comput. Stat.* **3**, 180–185 (2011).

[80] R Core Team. R: A language and environment for statistical computing. (2021).

[81] Anders, S. & Huber, W. Differential expression analysis for sequence count data. *Genome Biol.* **11**, R106 (2010). 20979621 10.1186/gb-2010-11-10-r106 PMC3218662

[82] Benjamini, Y. & Hochberg, Y. Controlling the False Discovery Rate: A Practical and Powerful Approach to Multiple Testing. *J. R Stat. Soc. Ser. B Methodol.* **57**, 289–300 (1995).

[83] Becker, M. G., Walker, P. L., Pulgar-Vidal, N. C., Belmonte, M. F. & SeqEnrich A tool to predict transcription factor networks from co-expressed Arabidopsis and Brassica napus gene sets. *PLOS ONE*. **12**, e0178256 (2017). 28575075 10.1371/journal.pone.0178256 PMC5456048

[84] Chan, A. & Belmonte, M. F. Histological and ultrastructural changes in canola (Brassica napus) funicular anatomy during the seed lifecycle. *Botany* **91**, 671–679 (2013).

