# Supplementary material for: Dual RNA sequencing reveals the transcriptomic and cellular response of Cannabis sativa to infection by the fungal pathogen Sclerotinia sclerotiorum
Source: Sci Rep. 2026 Apr 16;16:17739. doi: 10.1038/s41598-026-47998-2 (PMC13246782; doi:10.1038/s41598-026-47998-2)
Supplement: Supplementary file 2 — Supplementary Material 2 [file 41598_2026_47998_MOESM2_ESM.pdf]

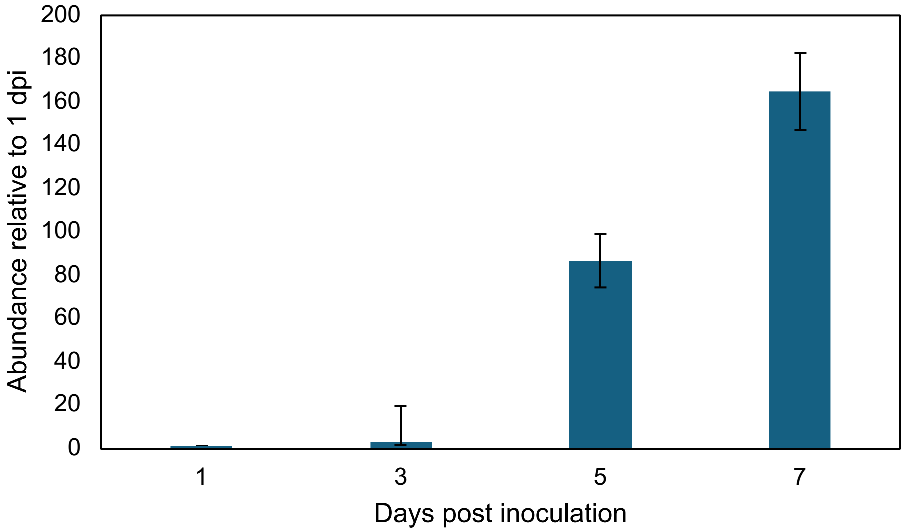

Figure S1. Relative fungal load of samples across time post inoculation determined by qPCR. *Sclerotinia sclerotiorum* 18S rDNA was used as a target for qPCR. Error bars correspond to standard error.

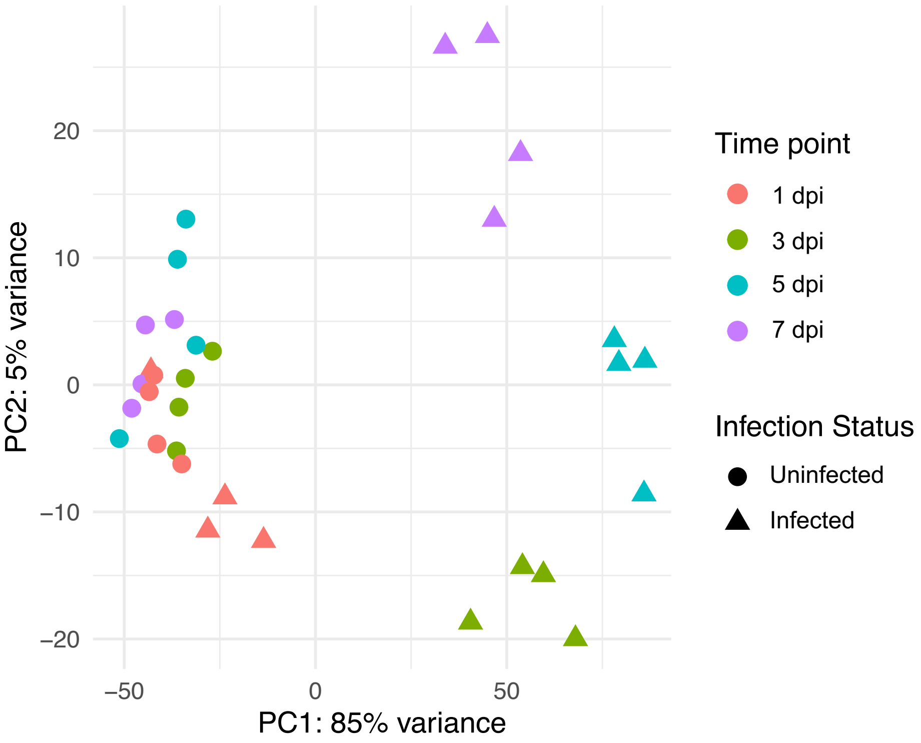

Figure S2. Principal component analysis (PCA) of *C. sativa* in the presence and absence of *S. sclerotiorum* across a seven-day infection period. Circles correspond to uninfected tissues, while triangles correspond to infected tissues. Colour corresponds to timepoint. PCA generated considering the top 1000 most variable genes. dpi = days post inoculation.

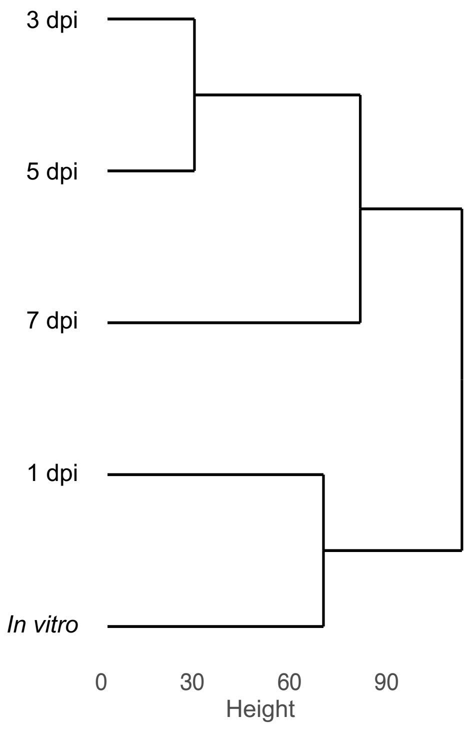

Figure S3. Hierarchical clustering of the top 1000 most variable genes of *S. sclerotiorum* grown *in vitro* or *in planta* during infection of *C. sativa*. Height corresponds to distance between clusters as computed by Euclidian distance. dpi = days post inoculation.

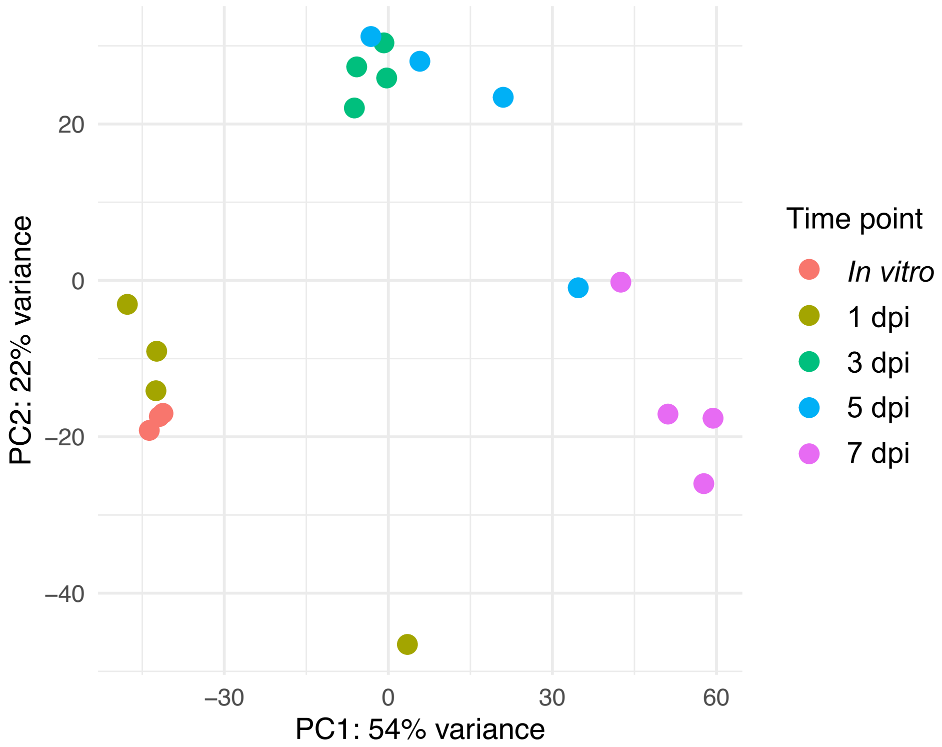

Figure S4. Principal component analysis (PCA) of *S. sclerotiorum* grown *in vitro* or *in planta* during a seven-day infection of *C. sativa*. Colour corresponds to timepoint, while red corresponds to *in vitro* grown *S. sclerotiorum*. PCA generated considering the top 500 most variable genes. dpi = days post inoculation.

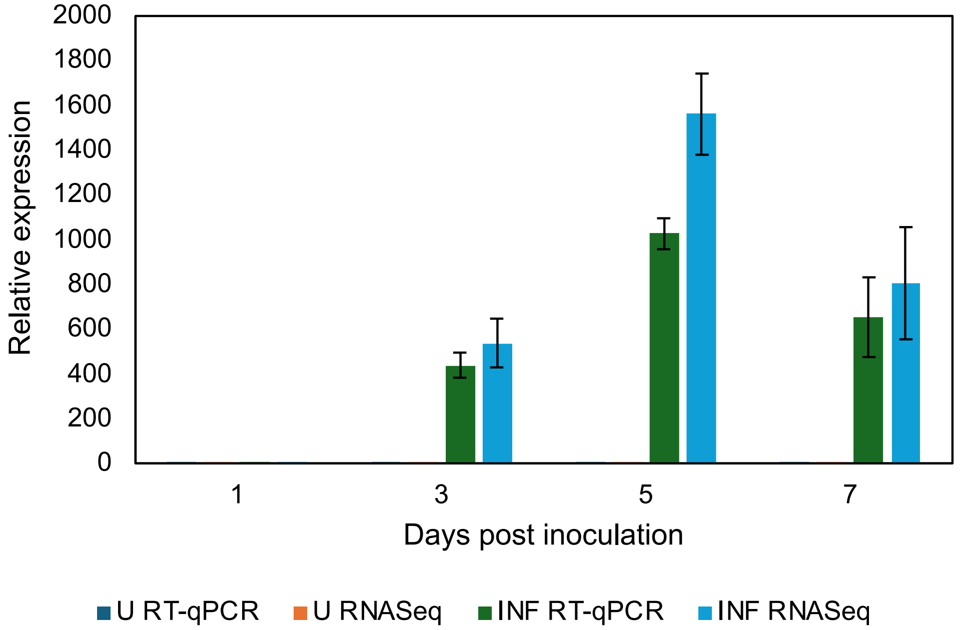

Figure S5. Comparison of relative expression levels of *PATHOGENESIS RELATED PROTEIN 1* resulting from RT-qPCR and RNA sequencing across time post inoculation with *Sclerotinia sclerotiorum*. U corresponds to uninfected samples (dark blue and orange), while INF corresponds to samples that had been inoculated with *S. sclerotiorum* (green and light blue). Bars correspond to standard error.

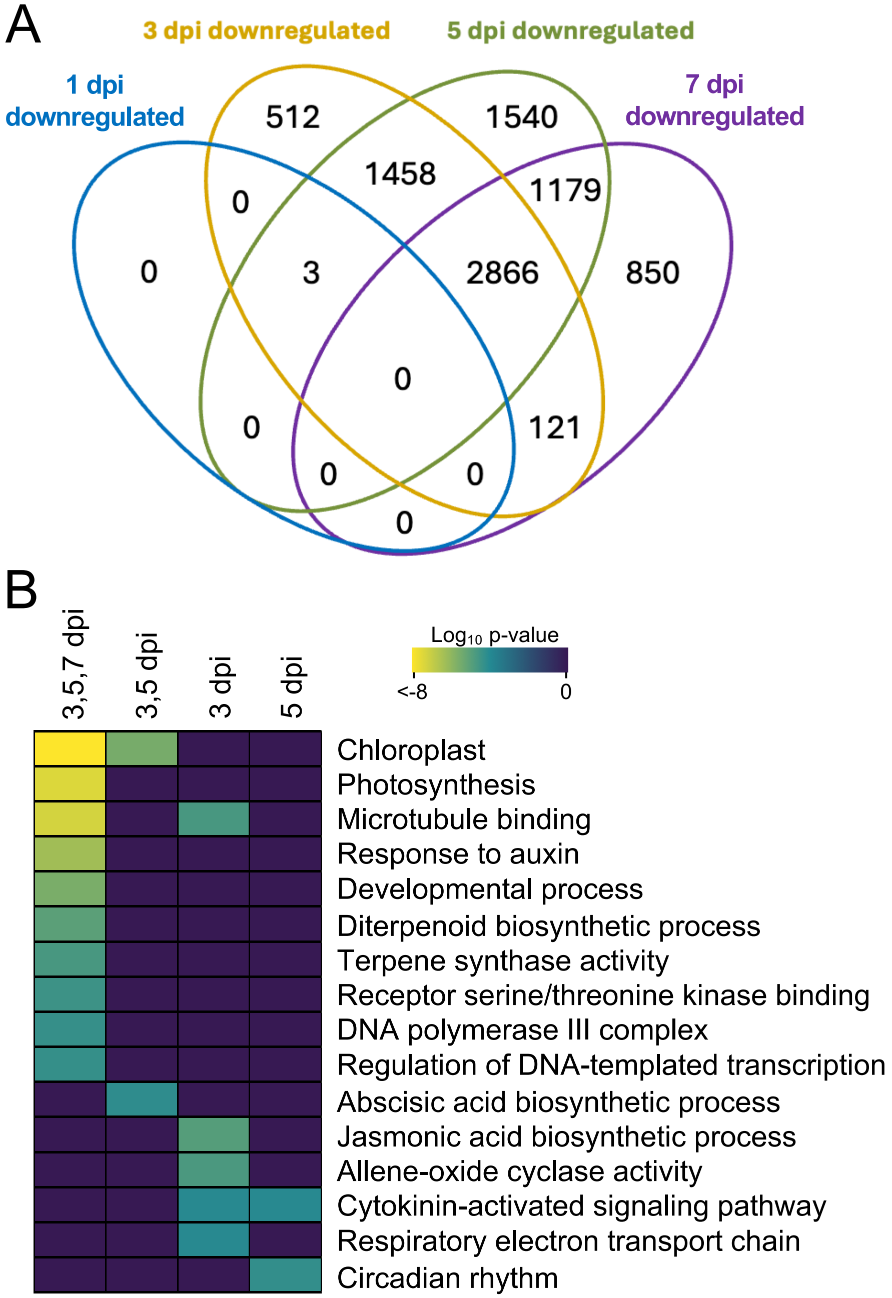

Figure S6. Differential gene expression analysis of down-regulated genes in *C. sativa* infected with *S. sclerotiorum* across a seven-day infection period. (A) Venn diagram of significantly downregulated differentially expressed gene sets (FDR<0.05) in response to infection. (B) Heatmap of significantly enriched GO terms (FDR <0.01) resulting from timepoint-specific and shared subsets. A brighter yellow colour indicates greater statistical significance. dpi = days post inoculation.

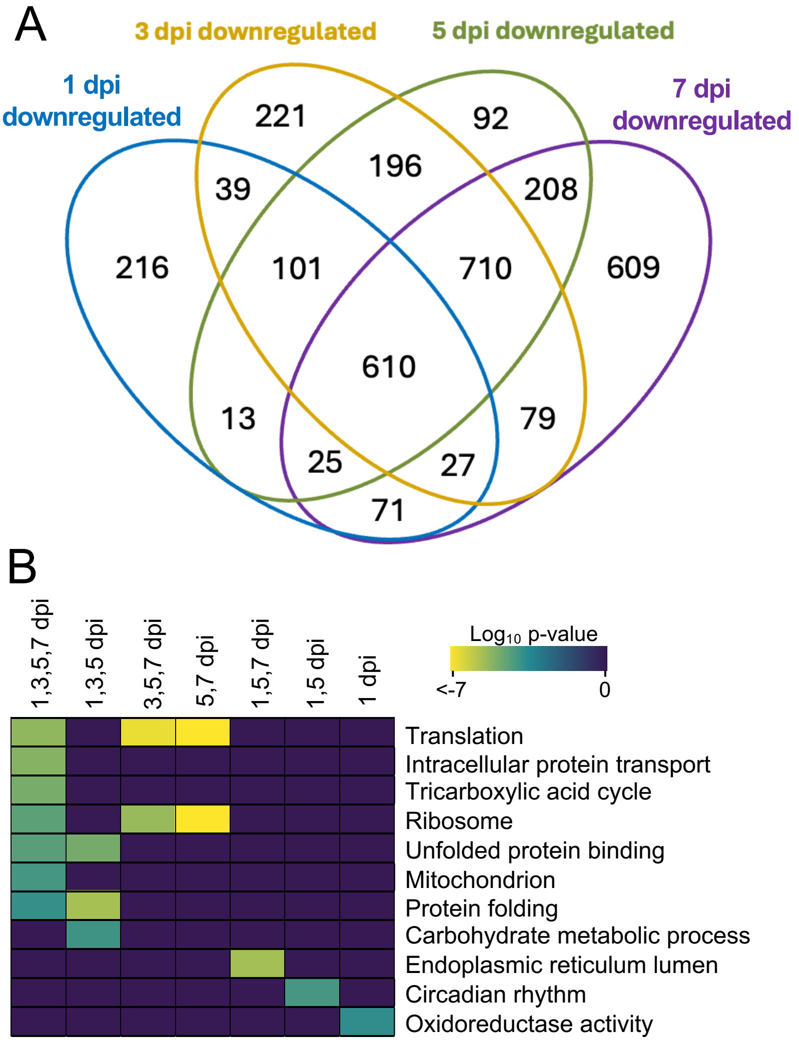

Figure S7. Differential gene expression analysis of significantly downregulated gene sets of *S. sclerotiorum* infection of *C. sativa* across a seven-day infection period. (A) Venn diagram of significantly downregulated differentially expressed gene sets (FDR<0.05) in response to infection. (B) Heatmap of significantly enriched GO terms (FDR <0.01) resulting from timepoint-specific and shared subsets. A brighter yellow colour indicates greater statistical significance. dpi = days post inoculation.

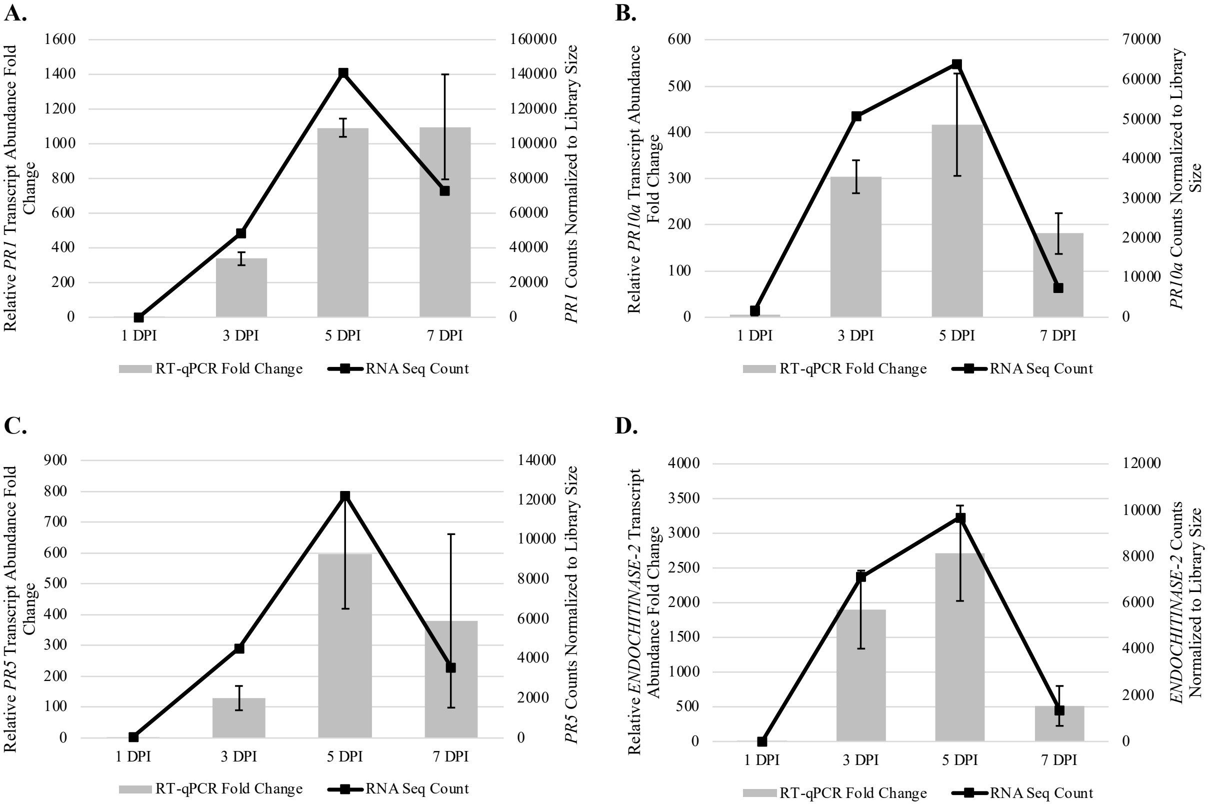

Figure S8. RT-qPCR validation of four representative *C. sativa* genes. A) *PATHOGENESIS RELATED PROTEIN 1* (*PR-1; LOC115704466*). B) *PATHOGENESIS-RELATED PROTEIN STH-2* (*PR-10a; LOC115722015*). C) *THAUMATIN-LIKE PROTEIN 1B* (*PR-5; LOC115710654*). D) *ENDOCHITINASE 2* (*LOC115705823*). A*DENINE PHOSPHO-RIOSYLTRANSFERASE 1* (*APT1*) was used as the housekeeping control. Target transcript relative abundance was calculated using the ΔΔCT method with four biological replicates quantified per timepoint. Error bars represent standard error. RNA sequencing gene counts were normalized to library size. Data were scored at 1,3, 5, and 7 days post inoculation (dpi). A complete list of primers sequences used in these experiments is found in Table S1.

Supplementary Table S1. Primer sequences, target information, and efficiencies of primers used in qPCR and RT-qPCR.

Species	Gene name/identifier	Sequence (5'-3')	Efficiency
S. sclerotiorum	18S rDNA	F: AGCCGATGGAAGTTTGAGGC<br>R: CTCGTTGGCTCTGTCAGTGT	104.8 %
C. sativa	*PATHOGENESIS RELATED PROTEIN 1*<br>LOC115704466	F: TTCGTCTTGGGTGTGCTAAG<br>R: ATCATATGGTCTCTGGCCATTC	102.4 %
	TIP41-LIKE PROTEIN<br>LOC115703022	F: GGCACCCAAAGAGCCTATTCT<br>R: CCCCATTATCTGCAAGTTCATCT	109.2 %
	ADENINE PHOSPHO-RIOSYLTRANSFERASE 1<br>LOC115713640	F: TTGCAACTGGAGGAACCTTGT<br>R: CATCCACTCCAACACGTTCAA	97.8 %
	PATHOGENESIS-RELATED PROTEIN STH-2 (PR-10a)<br>LOC115722015	F: ACTTCACTCAAGCTAGTCAGTTC<br>R: ACCCAATGGATCTCCTTCAATC	98.9 %
	THAUMATIN-LIKE PROTEIN 1B (PR-5)<br>LOC115710654	F: GCAACGGTGGTACTGGAAA<br>R: CATTGCTGACGTCGTAGTAGTC	100.8 %
	ENDOCHITINASE 2<br>LOC115705823	F: GGTGGGATCGAATGTGGTATAG<br>R: CGAACGACCTCTGGTTGTAA	100.5 %
